# Contribution of the 12–17 hydrophobic region of islet amyloid polypeptide in self-assembly and cytotoxicity

**DOI:** 10.3389/fmolb.2022.1017336

**Published:** 2022-10-03

**Authors:** Mathilde Fortier, Mélanie Côté-Cyr, Vy Nguyen, Margaryta Babych, Phuong Trang Nguyen, Roger Gaudreault, Steve Bourgault

**Affiliations:** ^1^ Department of Chemistry, Succursale Centre-Ville, Université du Québec à Montréal, Montreal, QC, Canada; ^2^ Quebec Network for Research on Protein Function, Engineering and Applications, PROTEO, Montreal, QC, Canada; ^3^ Department of Physics, Université de Montréal, Succursale Centre-ville, Montreal, QC, Canada

**Keywords:** islet amyloid polypeptide, self-assembly, amyloid fibrils, cytotoxicity, molecular dynamics simulation, conformation, peptide aggregation, secondary structure

## Abstract

The islet amyloid polypeptide (IAPP) is a 37-residue aggregation-prone peptide hormone whose deposition as insoluble fibrils in the islets of Langerhans is associated with type II diabetes. Therapeutic interventions targeting IAPP amyloidogenesis, which contributes to pancreatic β-cell degeneration, remain elusive owing to the lack of understanding of the self-assembly mechanisms and of the quaternary proteospecies mediating toxicity. While countless studies have investigated the contributions of the 20–29 amyloidogenic core in self-assembly, IAPP central region, *i.e.* positions 11 to 19, has been less studied, notwithstanding its potential key role in oligomerization. In this context, the present study aimed at investigating the physicochemical and conformational properties driving IAPP self-assembly and associated cytotoxicity. Computational tools and all-atom molecular dynamics simulation suggested that the hydrophobic 12–17 segment promotes IAPP self-recognition and aggregation. Alanine scanning revealed that the hydrophobic side chains of Leu12, Phe15 and Val17 are critical for amyloid fibril formation. Destabilization of the α-helical folding by Pro substitution enhanced self-assembly when the pyrrolidine ring was successively introduced at positions Ala13, Asn14 and Phe15, in comparison to respective Ala-substituted counterparts. Modulating the peptide backbone flexibility at position Leu16 through successive incorporation of Pro, Gly and α-methylalanine, inhibited amyloid formation and reduced cytotoxicity, while the isobutyl side chain of Leu16 was not critical for self-assembly and IAPP-mediated toxicity. These results highlight the importance of the 12–17 hydrophobic region of IAPP for self-recognition, ultimately supporting the development of therapeutic approaches to prevent oligomerization and/or fibrillization.

## Introduction

The islet amyloid polypeptide (IAPP) is a 37-residue hormone that is co-expressed and co-secreted with insulin by pancreatic β-cells (Milardi et al., 2021). Although its physiological functions are still not fully characterized, IAPP is known to inhibit glucagon secretion, food intake and gastric emptying (Hay et al., 2015). The coadministration of the IAPP analog pramlintide with insulin to diabetic patients contributes to normalizing glucose levels (Kruger and Gloster, 2004). Notwithstanding essential physiological functions and therapeutic usage, IAPP is known for its high propensity to aggregate into insoluble amyloid fibrils that are associated with the pathogenesis of type 2 diabetes (Westermark, 2011). The extracellular deposition of IAPP in the pancreatic islets is observed in over 90% of type 2 diabetic patients and correlates closely with the loss of function of pancreatic β-cells (Kahn, 2000). Different supramolecular structures of IAPP fibrils have been reported and have highlighted the high polymorphism of amyloid assemblies. As implied by solid-state NMR, each of the monomeric units adopt a U-shaped structure, with two β-strands connected by a chain-reversing loop involving residues 18-27 (Luca et al., 2007). In contrast, three recent Cryo-EM studies revealed that IAPP monomers within fibrils encompass three β-strands of various lengths (Cao et al., 2020; Gallardo et al., 2020; Röder et al., 2020). Interestingly, IAPP fibrils seeded with amyloids extracted from diabetic patients exhibit at least four different atomistic structures, suggesting that pancreatic islet deposits are heterogenous (Cao et al., 2021). This polymorphism complexifies our understanding of the relationships between amyloid deposition and β-cell degeneration, as different fibrillar architectures could lead to divergent toxicity (Nguyen et al., 2021). Furthermore, compelling biochemical evidence has revealed that oligomers and prefibrillar assemblies are the most cytotoxic proteospecies of the amyloidogenic cascade (Cao et al., 2013; Nguyen et al., 2015). Highly toxic IAPP oligomers transiently populating the lag phase were found to be soluble, globally flexible, partially structured with solvated aromatic side chains, and to range from dimers up to hexamers (Abedini et al., 2016). The large secondary and quaternary conformational ensemble of transient oligomers as well as the polymorphism of amyloid fibrils make it very challenging to determine the molecular basis of the toxicity of IAPP assemblies. Thus, it remains critical to elucidate the regions and the residue side chains driving self-recognition to ultimately design therapeutic strategies to arrest aggregation and IAPP-mediated cytotoxicity.

Countless studies have highlighted the key contributions of the 20–29 domain for IAPP self-assembly, often referred to as the amyloidogenic core (Glenner et al., 1988). The fact that the sequence from rodent IAPP (rIAPP) includes three β-breaker prolines within the 20-29 region, and is non-amyloidogenic and poorly toxic, further highlights the critical role of this segment (Moriarty and Raleigh, 1999; Tomasello et al., 2014). Short peptides comprising the 20-29 region spontaneously assemble into β-sheet-rich fibrillar structures (Westermark et al., 1990; Goldsbury et al., 2000; Azriel and Gazit, 2001; Babych et al., 2018). Intriguingly, while peptides corresponding to the highly conserved 8-20 central are known to self-assemble into fibrils (Jaikaran et al., 2001), a limited number of structure-aggregation studies have focused on this region. It was reported that the minimal peptide sequences of the 8–20 domain competent to self-associate into amyloids consist of the segments 12-17 and 15-20 (Scrocchi et al., 2003). Besides, residue-specific modifications have been exploited to refine the driving forces of IAPP self-assembly (Cao et al., 2013). While the critical roles of several side chains of the 20-29 region for aggregation have been reported, including residues Asn21 (Koo et al., 2008; Godin et al., 2019), Phe23 (Doran et al., 2012; Tu and Raleigh, 2013), Ser28 (Westermark et al., 1990; Ridgway et al., 2020) and Ser29 (Akter et al., 2018; Ridgway et al., 2020), other segments of IAPP have been less investigated. Within the 8-20 region, Asn14 is likely involved in a critical hydrogen bond network favoring IAPP nucleation (Koo et al., 2008; Nguyen et al., 2017), whereas the hydrophobic Phe15 is contributing in IAPP oligomerization (Wiltzius et al., 2009; Tu and Raleigh, 2013). These previous structure-aggregation studies have demonstrated that the process of self-assembly is complex and involves molecular interactions between multiple domains of IAPP, illustrating the importance of conducting such investigation with the full-length peptide.

The present study aimed at further deciphering the physicochemical and conformational properties of IAPP contributing to amyloid aggregation and associated cytotoxicity. *In silico* analyses performed with AGGRESCAN (Conchillo-Solé et al., 2007), CamSol (Sormanni et al., 2015) and TANGO (Fernandez-Escamilla et al., 2004) predicted two main regions respectively contributing to aggregation, low solubility and β-sheet formation: segments 12-17 and 23-29. Furthermore, all-atom molecular dynamics simulation indicated that the 12-17 central domain of IAPP participates in peptide self-recognition. While the 23–29 domain has been extensively studied, the segment 12–17 has been less investigated. After demonstrating that a short peptide corresponding to the 12–17 domain assembled into fibrillar nanostructures, the importance of each side chain in amyloid formation and cytotoxicity was investigated by successively substituting each residue with Ala (*Ala-scan*). By replacing residues within this segment by amino acids affecting the secondary conformational ensemble, such as Pro, Gly and aminoisobutyric acid (Aib), insights into the mechanisms of amyloid formation were obtained. Our results revealed the critical contributions of the hydrophobic sidechains of Leu12, Phe15 and Val17 in self-assembly as well the importance of backbone flexibility and local secondary structure for amyloid formation.

## Materials and methods

### Molecular dynamics

The solution structure of monomeric IAPP determined by NMR in presence of sodium dodecyl sulfate (SDS) micelles (PDB 2L86) (Nanga et al., 2011) was converted to be compatible with GROMACS, using ACPYPE (Sousa da Silva and Vranken, 2012). The starting distance between monomers was the equivalent of the size of a monomer. IAPP homodimer molecular system was prepared with the following steps: 1) the system underwent an energy minimization in vacuum using the steepest descent (SD) and conjugate gradient (CG) algorithms; 2) the system was loaded into a periodic dodecahedron box, which was then solvated with water molecules (TIP3P) (distance from the wall: 1 nm); 3) ions were added to the system to ensure neutrality; 4) the system underwent a second energy minimization using the SD and CG algorithms while restraining all non-hydrogen atoms with harmonic restraints; 5) the system was equilibrated in the NVT ensemble at 300 K over 10 ns while maintaining the previous harmonic restraints; 6) the system was equilibrated in the NPT ensemble at 1 bar over 10 ns while maintaining the previous harmonic restraints; and 7) the system underwent a full molecular dynamics (MD) simulation without any harmonic restraints. All simulations were run with GROMACS with the AMBER99sb-star-ildnp forcefield (Aliev et al., 2014). The system was neutralized with the addition of counter ions (6 Cl^-^ for IAPP homodimer) and the Nosé–Hoover thermostat was used with a coupling constant of 0.1 ps to maintain the temperature constant at 300 K (Hoover et al., 1985). The Parrinello–Rahman barostat was used with a coupling constant of 2.0 ps to maintain the pressure constant at 0.987 atm. A cutoff of 1 nm was applied to both van der Waals and electrostatic interactions, the latter being computed using Particle Mesh–Ewald and LINCS and SETTLE were used to constrain bond lengths and water geometry, respectively. The generalized AMBER forcefield (GAFF) (Wang et al., 2004) was used to determine the parameters of the peptides and the RESP protocol from ANTECHAMBER (Wang et al., 2006) was used to determine the partial charges of the peptides. A 1000-ns MD simulation was launched on IAPP homodimer system according to the above protocol. The MD trajectory was analyzed by means of several variables, including root mean square deviation (RMSD), root mean square fluctuation (RMSF), hydrogen bond (H-bond), solvent accessible surface area (SASA), secondary structures, average minimum distances, residue-pairwise distance maps (mdmat) and probability of contacts. The analysis of MD simulations was performed using built-in GROMACS tools and in-house scripts. The RMSD and the RMSF were analyzed to determine conformational changes and to determine the convergence interval. H-bonds were defined using a 0.35 nm distance cut-off and a 30° hydrogen–donor–acceptor angle cut-off. The SASA was computed over the 1,000 ns trajectory. Secondary structures were determined using DSSP (Kabsch and Sander, 1983) and the average probability of secondary structures were analyzed over the last 500 ns trajectory to avoid potential bias induced from the initial state. Using pairdist, the average minimum distances of every residue of peptide A with the entire peptide B as well as of every residue of peptide B with the whole peptide A, were calculated. Using mdmat, the residue-pairwise distance maps between peptide A with peptide B were calculated every 50 ns over the interval of 500 to 1,000 ns. From these pairwise maps, and assuming the probability of a contact occurs when the distance between interchain residues is <0.6 nm, the interchain pairwise residue contact probability was determined. PyMOL molecular graphics system was used for the visualization of molecules and of the MD trajectory (The PyMOL Molecular Graphics System, Version 2.5; Schrödinger, LLC. NewYork, NY, United States , 2021).

### Peptide Synthesis, Purification and Monomerization

IAPP and its derivatives were synthesized on Rink Amide AM solid support using fluorenylmethyloxycarbonyl chloride (Fmoc) chemistry (De Carufel et al., 2013). Pseudo-proline derivatives were used to facilitate synthesis and to increase the yield (Abedini and Raleigh, 2005). After cleavage from the resin with a mixture of TFA, ethanedithiol, phenol, and water, crude peptides were precipitated with ethyl ether, solubilized in H_2_O, and lyophilized. Peptides were first dissolved in acetic acid and diluted in H_2_O to 35% to be purified by RP-HPLC using a C_18_ column. Collected fractions were analyzed by mass spectrometry using a LC-ESI-TOF. Fractions corresponding to the desired peptide were pooled and oxidation of Cys2 and Cys7 was performed in dimethyl sulfoxide (DMSO) overnight for 16 h. Cyclized peptides were then diluted in 0.06% TFA water before being purified by RP-HPLC. Fractions with purity higher than 95% were pooled and lyophilized. Monomerized IAPP were prepared by dissolving the lyophilized peptide in 100% hexafluoro-2-propanol (HFIP) to a concentration of 1 mg/ml. Solution was filtered through a 0.22-μm hydrophilic polyvinylidene difluoride (PVDF) filter and sonicated for 30 min before being lyophilized. The resulting powder was solubilized at 1 mg/ml for a second time in HFIP, sonicated for 30 min, and solution was aliquoted and lyophilized. Monomerized IAPP samples were kept dried at -20°C until used.

### Kinetics of Amyloid Formation Monitored by ThT Fluorescence

Aggregation mixtures were prepared by dissolving the lyophilized peptide at a concentration of 50 μM in 20 mM Tris-HCl, pH 7.4. Assays were performed at 25°C without stirring in sealed black-wall clear-bottom 96-well nonbinding surface plates with 100 μL/well. Final peptide concentrations were 12.5 and 25 μM, and thioflavin T (ThT) concentration was fixed at 40 μM. Fluorescence was measured every 10 min (excitation: 440 nm, emission: 485 nm) and data from triplicate wells were averaged, corrected by subtracting the blank control, and plotted as ThT fluorescence vs*.* time. Data were normalized with maximum ThT fluorescence intensity obtained for unmodified IAPP. Data were fitted to a sigmoidal Boltzmann model:

$$Y=\frac{Yo+\left(Ymax-Yo\right)}{1+e^{-\left(T-T50\right)}}/\;K$$

where *T*_50_ is the time required to reach half of the maximum fluorescence intensity, *k* is the apparent first-order constant, and *Y*_max_ and *Y*_0_ are the maximum and initial ThT fluorescence values. Lag time corresponds to T_50_ - 2/*k*. Data of at least six assays performed with different peptide lots were averaged and expressed as the mean ± S.D. Statistical analysis was performed using Student’s *t* test, and statistically significant difference between IAPP and analogs was established at (*) *p* < 0.05; (**) *p* < 0.005; (***) *p* < 0.0005.

### Peptide self-assembly

Solutions were prepared by dissolving the peptide at a concentration of 50 μM in 20 mM Tris-HCl, pH 7.4, and were incubated at room temperature without agitation in 1.5 ml *Eppendorf* microtubes. For IAPP_12-17_, the lyophilized peptide was dissolved in 20 mM acetate pH 6.5 in the presence of 3% DMSO, or HFIP, at a final concentration of 500 μM and the peptide solution was incubated at 37 °C under constant rotational agitation.

### ThT and ANS fluorescence spectroscopy

At the indicating time of incubation, peptide solutions were diluted to 25 μM in presence of 40 μM ThT, or 25 μM 8-anilino-1-naphthalenesulfonic acid (ANS). Fluorescence was measured in 10-mm pathlength microcells using a PTI QuantaMaster spectrofluorometer. For ThT, excitation was set at 440 nm, and the emission spectrum from 450 to 550 nm was recorded. For ANS, the excitation was set at 355 nm, and the emission scan was recorded from 385 to 585 nm.

### Circular dichroism spectroscopy

After incubation, peptide solutions were diluted in water to 25 μM and incorporated into a 2-mm path length quartz cell. For IAPP_12-17,_ the solution was diluted to 150 μM. Far-UV CD spectra were recorded from 190 to 260 nm using a Jasco J-815 CD spectropolarimeter at 25°C. The wavelength step was set at 0.5 nm with an average time of 10 s per scan at each wavelength. Spectra were background-subtracted with buffer solution. Raw data were converted to mean residue ellipticity (MRE):

$$MRE=\frac{Mean\;residue\;weight\;\left(\frac{g}{mol}\right)X\;CD\;signal\;\left(deg\right)}{10\;X\;pathlength\;\left(cm\right)\;X\;peptide\;concentration\;\left(\frac{g}{mL}\right)}$$

### Transmission electron microscopy

After incubation, peptide solutions were diluted to 10 μM, or 100 μΜ for IAPP_12-17_, before being applied on glow-discharged carbon films on 300-mesh copper grids. Samples were adsorbed and negatively stained with 1.5% uranyl formate for 45 s. Images were recorded using a FEI Tecnai G2 Spirit Twin microscope operating at 120 kV and equipped with a Gatan Ultrascan scan 4,000 4k × 4k CCD camera system.

### Atomic force microscopy

After incubation, peptide solutions were diluted in 1% acetic acid to a concentration of 7.5 μΜ. Samples were immediately applied to freshly cleaved micas, washed twice with deionized water and air-dried for 24 h. Images were recorded using Veeco/Bruker multimode AFM instrument using ScanAsyst-air mode with a silicon tip (2–12 nm tip radius, 0.4 N/m force) on a nitride lever. Images were taken at 0.977 Hz and 512 scan/min.

### Lipid membrane leakage

Large unilamellar vesicles (LUVs) were assembled from 1,2-Dioleoyl-sn-glycero-3-phosphocholine (DOPC) and 2-dioleoyl-sn-glycero-3-phospho-(1′-rac-glycerol) (DOPG) at a 9:1 M ratio (DOPC:DOPG). Lipids were solubilized in 100% chloroform, which was then evaporated. The lipid film was rehydrated in 20 mM Tris-HCl pH 7.4 containing 70 mM calcein, whose solubility was increased by the addition of 5M NaOH and sonication. The lipid mixture was freeze-thawed five times before being extruded through a 100 nm membrane for 20 cycles. Nonencapsulated calcein was removed by gel filtration using a Sephadex G25 fine resin and 20 mM Tris-HCl, 150 mM NaCl, pH 7.4, as elution buffer. Final lipid concentration was determined by a colorimetric assay (Babych et al., 2021). Lyophilized peptides were solubilized in 20 mM Tris-HCl, 150 mM NaCl, pH 7.4, at 50 μM prior to dilution into calcein-LUVs, to reach 25 μM peptide and 500 μM phospholipids. Fluorescence was monitored in sealed black-wall, clear-bottom 96-well non-binding surface plates with 100 μL/well. Fluorescence (excitation: 495 nm, emission: 517 nm) was measured every 10 min over 24 h. 100% leakage (F_max_) was determined with 0.2% Triton X-100 and LUV leakage was reported using:

Membrane leakage (%) = (*F*−*F*_baseline_)/(*F*_max_−*F*_baseline_)

Data of at least four assays with different peptide lots were averaged and expressed as the mean ± S.D. Statistical analysis was performed using Student’s *t* test, and statistically significant difference was established at (*) *p* < 0.05; (**) *p* < 0.005; (***) *p* < 0.0005.

### Measurement of cell viability

Rat INS-1E cells were cultured in treated black-wall clear-bottom 96-well plates at a density of 25,000 cell/well in RPMI-1640 medium supplemented with 10% FBS, 2 mM l-glutamine, 100 U/ml penicillin, 100 μg/ml streptomycin, 10 mM HEPES, 1 mM sodium pyruvate and 50 μM β-mercaptoethanol. After 48 h of incubation at 37°C in 5% CO_2_, cells were treated with 50 μL of peptide freshly dissolved in 20 mM Tris-HCl, pH 7.4. Cells were incubated with the peptide for 24 h before cell viability was measured with the reduction of 45 μM resazurin. Cell viability (%) was calculated from the ratio of the fluorescence of the treated cells to the vehicle-treated cells. Data of at least five assays performed with different peptide lots were averaged and expressed as the mean ± S.D. Statistical analysis was performed using Student’s *t* test, and statistically significant difference was established at (*) *p* < 0.05; (**) *p* < 0.005; (***) *p* < 0.0005. For Live/Dead viability assay, cells were plated in 12-well plates at density of 250,000 cell/well for 48 h before treatment with 25 μM peptide. After 24 h incubation, 4 μM of ethidium homodimer-1 and 2 μM of calcein-AM were added to the media, and after 30 min of incubation, cells were imaged with a fluorescence confocal Nikon microscope equipped with a ×20 objective.

## Results and discussion

### The fragment Leu12-Val17 self-assembles into β-sheet-rich fibrils

**FIGURE 1:**
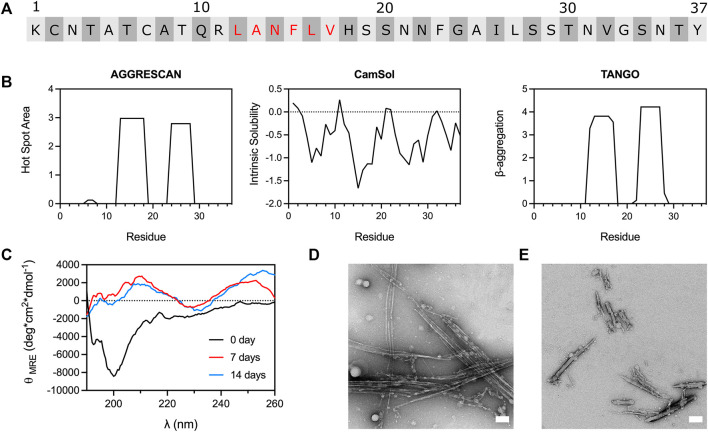
Amyloidogenicity of the 12–17 region of IAPP. **(A)** IAPP primary sequence with the residues of the 12–17 segment indicated in red. **(B)** Computational predictions performed with AGGRESCAN, CamSol and TANGO, carried out with amidated C-terminus. **(C)** CD spectra of IAPP_12-17_ over incubation time. IAPP_12-17_ peptide was solubilized at 500 μM in 20 mM acetate buffer, pH 6.5, 3% HFIP, and incubated at 37 °C under constant agitation. **(D,E)** TEM images of IAPP_12-17_ peptide after 7 days incubation at 500 μM in presence of **(D)** 3% HFIP and **(E)** 3% DMSO. Scale bars: 100 nm.

Analysis of IAPP sequence with the *in silico* tools AGGRESCAN (Conchillo-Solé et al., 2007), CamSol (Sormanni et al., 2015) and TANGO (Fernandez-Escamilla et al., 2004) revealed two main amyloidogenic regions: the 12-17 and the 23–29 segments (Figure 1), with the 12-17 region predicted to be the most aggregation-promoting region. Whereas the implication of the 23-29 region in amyloid formation has been abundantly investigated (Goldsbury et al., 2000; Buchanan et al., 2013; Wang et al., 2014), the segment 12–17 has been the subject of a limited number of studies (Scrocchi et al., 2003; Kajava et al., 2005). In this context, we initially evaluated if a short C-amidated peptide encompassing the 12–17 segment (LANFLV) could assemble into amyloid fibrils. As the IAPP_12-17_ peptide showed poor solubility, the lyophilized/monomerized peptide was first solubilized in HFIP or DMSO, before being diluted in 20 mM acetate buffer pH 6.5 to reach a final concentration of 3% of organic solvent and 500 μM peptide. The LANFLV hexapeptide was incubated at 37 °C under constant rotary agitation and periodically analyzed by ThT and ANS fluorescence, CD spectroscopy, and TEM. Far-UV spectroscopy revealed that IAPP_12-17_ exhibits mainly a random coil secondary structure after its solubilization, which evolves into β-sheet after prolonged incubation times, suggesting the formation of amyloid-like assemblies (Figure 1C). Because of high signal background, CD analysis was not performed in presence of DMSO. TEM imaging performed after 7 days incubation exposed the formation of laterally aligned rod-like fibrils, which were longer in presence of HFIP, in comparison to DMSO (Figures 1D,E). These β-sheet-rich fibrillar aggregates were ThT and ANS negative (Supplementary Figure S1), which could be attributed to the fact that this segment might not be sufficient to generate π-stacking and/or large hydrophobic surfaces (Biancalana and Koide, 2010). In a recent study, a pentapeptide corresponding to the segment 13-17 (ANFLV) did not form fibrils, even after several days of incubation (Rozniakowski et al., 2020), suggestive of the critical role of Leu12. Nonetheless, as amyloid self-assembly is very sensitive to the experimental conditions (Khemtémourian et al., 2011), such as the presence of organic solvents (Padrick and Miranker, 2002; Nichols et al., 2005), comparison between studies remains problematic. Overall, these data indicate that a short peptide comprising the predicted aggregation-prone 12-17 region forms fibril-like nanostructures, encouraging further investigation to elucidate its contributions to the self-assembly of full-length IAPP.

### Molecular dynamics exposes residues contributing to IAPP self-recognition

**FIGURE 2:**
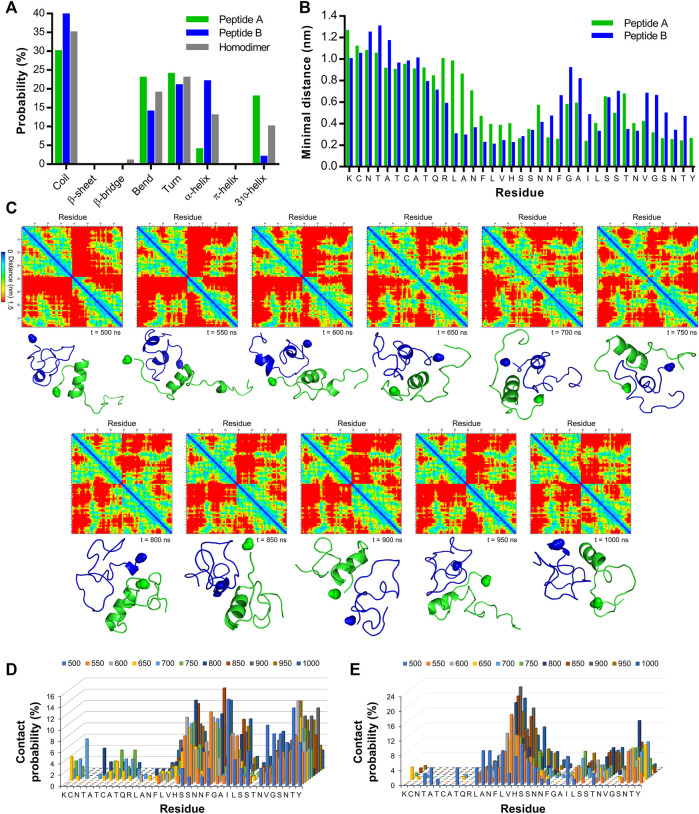
All-atom molecular dynamics simulation of IAPP self-recognition **(A)** Average secondary structure probability in IAPP homodimer and discrete monomers. **(B)** Average minimal distances for each residue of peptide A with the entire peptide B as well as for each residue of peptide B with the entire peptide **(A) (C)** Interchain pairwise (residue-residue) distance maps for IAPP homodimer and corresponding cartoon representation of IAPP dimer at every 50 ns over the 500–1,000 ns interval. The C-terminal amide group is highlighted as a sphere. Peptide A is represented in green and peptide B in blue **(D,E)** Probability of interchain pairwise (residue-residue) contacts (<0.6 nm distance) for every 50 ns over the 500–1,000 ns interval for **(D)** peptide A and **(E)** peptide **(B)**.

All-atom molecular dynamics was used to probe the residues participating in IAPP self-recognition. Two monomers (PDB 2L86) were placed into a periodic decahedron box (10.3 nm × 10. nm × 7.3 nm) in presence of 25,165 water molecules. The monomers A and B were manually positioned at an interchain distance of 3 nm, and their evolution was computed over 1,000 ns at 300 K using the AMBER99sb-star-ildnp force field. The RMSD readily reached a plateau after 100 ns and was stable over the rest of the 1,000 ns trajectory (Supplementary Figure S2A). The RMSF of each Cα of peptides A and B, as well as the average H-bond number showed a similar stability over the 1,000 ns trajectory (Supplementary Figure S2B, C). The SASA was unstable at the beginning of the trajectory and became stable in the last 500 ns trajectory with an average of 52.9 nm^2^ (Supplementary Figure S1D). The number of secondary structures indicated a stable, albeit fluctuating and dynamic, homodimer over the same interval. Based on these observations, we selected the 500–1,000 ns interval to interrogate the secondary structures, the average minimum distances, and the probability of interchain contacts. Calculations showed that IAPP homodimer has a minimal β-sheet probability over the 500 ns trajectory (Figure 2A, Supplementary Figure S3), in agreement with previous studies proposing that the early oligomers are mainly disordered (Li et al., 2021). In fact, the predominant secondary structure is the random coil, followed by turn, bend, α-helix and 3_10_-helix. It is worth mentioning that the occurrence of the secondary structures as a function of time for monomer A did not mirror the one observed for monomer B. For instance, while peptide B showed 22% probability of α-helix, this probability was 4% for peptide A. This observation agrees with the recently proposed symmetry-breaking theory proposing a distribution of partially ordered and disordered monomers in the early steps of protein self-assembly (La Rosa et al., 2020). The average minimal interchain distances between monomers also revealed the asymmetry between the two IAPP molecules, with the 12-19 region of peptide B being actively involved in self-recognition and the central and C-terminal domains of monomer A contributing to interchain interactions (Figure 2B; Supplementary Figure S4). The interchain distances between each residue of each monomer were plotted every 50 ns over the 500–1,000 ns interval. Interchain pairwise (residue-residue) contact maps showed the evolution of the binding areas involved in the interactions between IAPP molecules with the distances from corresponding residues in the *z* direction; from 0 nm in blue to 1.5 nm in red (Figure 2C). The segment 12-17 of peptide B was actively implicated in interchain binding. In contrast, dimerization of peptide A implicated the participation of its C-terminal domain. The cartoon representations of the homodimer structure overtime are shown under each of the residue-pairwise contact maps (Figure 2C). In agreement with the probability of secondary structures (Figure 2A), the 3_10_-helix conformation of peptide A persisted over time in most snapshots, involving mainly the S19-S28 region of IAPP (Figure 2C). Moreover, residue-pairwise intrachain distance maps of IAPP discrete monomers for peptides A and B are shown at 500, 750 and 1,000 ns (Supplementary Figure S5). The probability of contacts was calculated from the number of contacts (<0.6 nm) for each 50 ns over the 500–1,000 ns interval, and this analysis revealed that interchain interactions are mainly occurring in the central and C-terminal regions for monomer A (segments 19-28 and 32-37), and in the 12-18 and 35–37 segments for monomer B (Figures 2D,E; Supplementary Figure S6). For peptide A, the residues showing the highest average probability of interchain contacts were Ser19 (9.2%), Phe23 (9.9%), Ile26 (8.0%) and Thr36 (8.9%) (Figure 2D). In sharp contrast, the residues Phe15, Leu16, Val17 and His18 of peptide B showed high probability of interchain contacts (Figure 2E). It has been recently shown by molecular dynamics simulation that these residues have large contact numbers in IAPP homodimers (Li et al., 2021), whereas residues Phe15, His18, Phe23 and Tyr37 were reported critical for IAPP aggregation (Padrick and Miranker, 2001; Luca et al., 2007). As previously reported, molecular dynamics simulation revealed that the contribution of the N-terminal region to IAPP self-recognition is exceedingly low (Jaikaran et al., 2001; Andreetto et al., 2010). These observations agree with a previous study based on a biarsenical fluorogenic probe that revealed rapid IAPP monomer self-recognition through the convergence of C-terminal domains, while the N-terminal domains only come close to each other upon the formation of the cross-β-sheet amyloid structure (Quittot et al., 2018). Overall, all-atom molecular dynamics simulation indicates that the 12–17 domain of IAPP participates in self-recognition, claiming for further investigation.

### Ala-scan reveals key contributions of residue side chains of the 12-17 domain for amyloid formation

**FIGURE 3:**
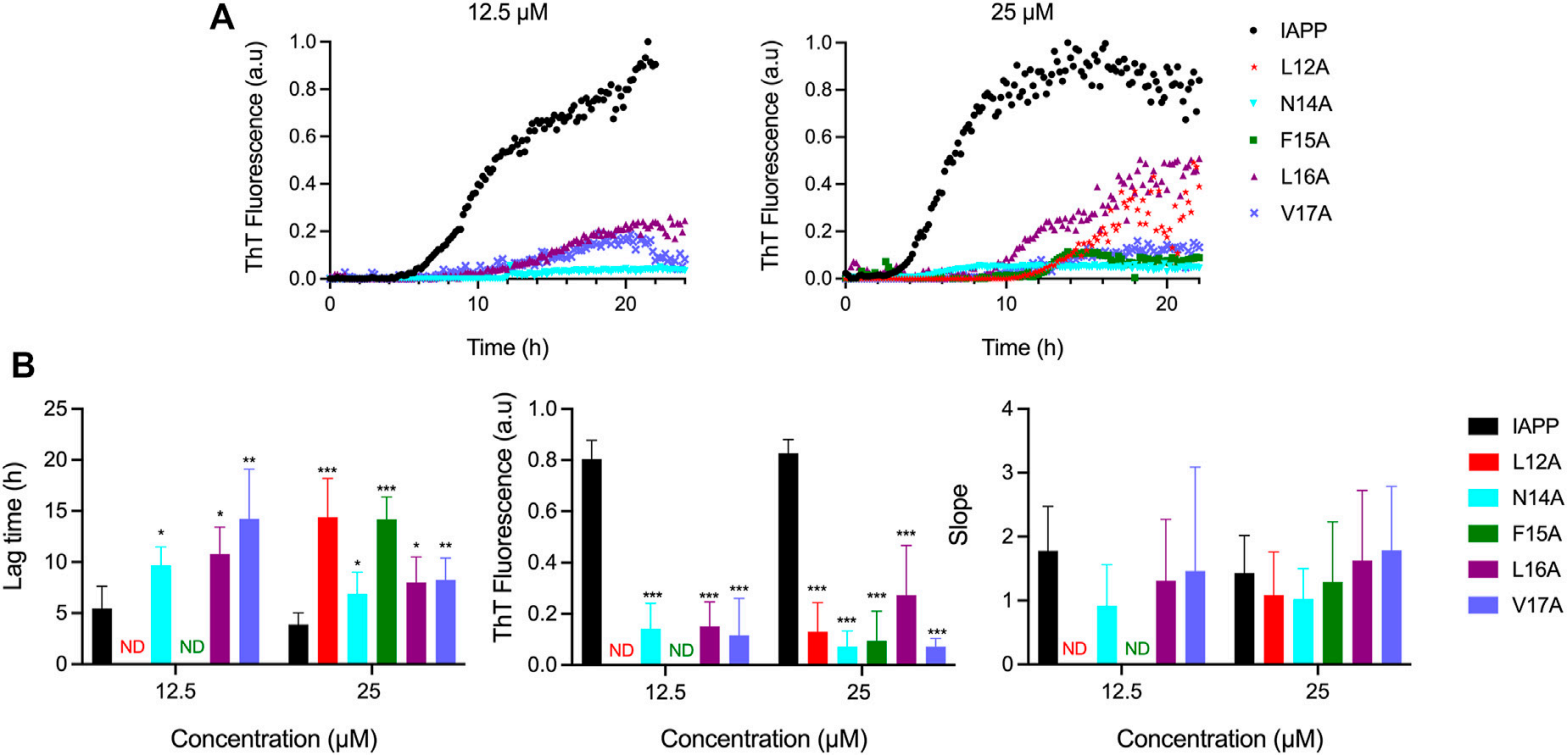
Effects of Ala substitution within the 12–17 domain on the kinetics of IAPP self-assembly. **(A)** Representative kinetics of amyloid formation monitored by ThT fluorescence at a peptide concentration of 12.5 and 25 μM. IAPP and its derivatives were incubated in 20 mM Tris-HCl, pH 7.4, and fluorescence was measured every 10 min. Data obtained from triplicate wells were averaged, corrected by subtracting the vehicle control, and plotted as ThT fluorescence vs*.* time **(B)** Parameters extracted from ThT kinetic traces. Data represent mean ± S.D. of a least six experiments performed in triplicate and significant difference between IAPP, and analogs was established at (*) *p* < 0.05; (**) *p* < 0.005; (***) *p* < 0.0005.

As revealed by *in silico* analysis, molecular dynamics simulation and biophysical analysis of a short peptide fragment, the 12–17 segment of IAPP likely contributes to IAPP self-recognition, although this region has been poorly studied so far. Because of challenges associated with the synthesis and handling of full-length IAPP, previous studies have often been conducted using short fragments (Scrocchi et al., 2003; Wiltzius et al., 2008; Louros et al., 2015; Rozniakowski et al., 2020), or the IAPP_8-37_ segment (Abedini and Raleigh, 2006; Koo et al., 2008; Wiltzius et al., 2009), limiting the scope, as amyloid formation implicates multiple domains of the peptide, as observed herein by molecular dynamics simulation (Figure 2). To initially probe the contributions of the physicochemical properties of each side chain of the 12-17 region in self-assembly and cytotoxicity, Ala was successively incorporated at positions 12 to 17. *In-silico* analysis predicted that the respective replacement of the hydrophobic residues Leu12, Phe15, Leu16 and Val17 by an Ala, decreases IAPP amyloidogenicity, while the N14A substitution increases its aggregation propensity (Supplementary Figure S7). Kinetics of amyloid formation monitored by ThT fluorescence revealed that the successive alanine replacement within the 12–17 domain inhibits, or delays, amyloid formation (Figure 3). Under the conditions of the kinetics assay, *i.e.* 20 mM Tris-HCl, pH 7.4, no agitation, nonbinding surface, unmodified IAPP showed a prototypical sigmoidal curve characteristic of a nucleated self-assembly process with a lag time of 3.9 ± 1.2 h at 25 μM (Figure 3B). In sharp contrast, the analogs L12A and F15A displayed a delayed lag time of respectively 13.5 ± 4.1 h and 14.2 ± 2.2 h, indicative of a key contribution of Leu12 and Phe15 in nucleation. At 12.5 μM concentration, no increase of ThT fluorescence signal could be detected for these two analogs. All alanine substitutions reduced considerably the final ThT fluorescence, suggesting either a reduction of the number of amyloids and/or a decrease of consecutive β-strands in the cross-β-sheet quaternary motif (Biancalana and Koide, 2010; Xue et al., 2017).

**FIGURE 4:**
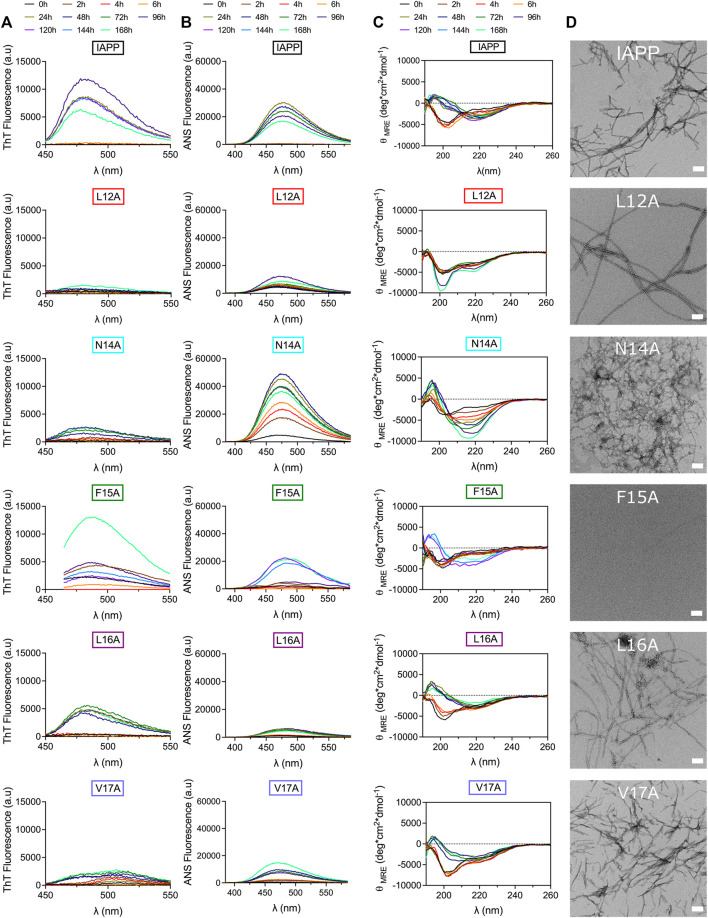
Effect of Ala-substitution within the 12–17 segment of IAPP on amyloid formation. **(A–D)** Peptides were incubated at 50 μM under fully quiescent conditions in 20 mM Tris-HCl buffer, pH 7.4, and periodically analysed by **(A)** ThT fluorescence, **(B)** ANS fluorescence, and **(C)** CD spectroscopy. **(D)** TEM images after 48 h incubation. Scale bar: 100 nm.

Considering that the frequent displacements of the microplate within the fluorimeter induce considerable agitation, which promotes amyloid formation through primary (mass transport) and secondary (fibril fragmentation) nucleation (Grigolato et al., 2017; Sebastiao et al., 2017), self-assembly was evaluated under fully quiescent conditions. IAPP and Ala-substituted analogs were incubated at 50 μM in 20 mM Tris-HCl, pH 7.4 in 1.5 ml microcentrifuge tubes and peptide solutions were periodically evaluated by far-UV CD spectroscopy, ThT and ANS fluorescence, TEM, and AFM. Under these conditions, unmodified IAPP shifted from a random coil (minimum around 200 nm) to a β-sheet secondary conformation (minimum at 218 nm) after 24 h of incubation, which was associated with an increase of ThT and ANS fluorescence, and the formation of prototypical fibrils (Figure 4). In sharp contrast, the L12A derivative remained mainly under a random coil structure after a week of incubation, which was associated with an absence of a ThT-signal and low ANS fluorescence. Surprisingly, long and twisted fibrils were observed by TEM after 48 h incubation (Figure 4D), indicating that the Leu→Ala substitution at position 12 induces the formation of assemblies characterized with a poorly defined cross-β quaternary organization. The N14A modification induced the appearance of CD spectra characteristic of a helical-like conformation with two minima at 206 and 222 nm between 2 and 24 h incubation, which shifted towards β-sheet after 48 h. TEM analysis revealed a dense network of thin fibrils after 48 h incubation, which were associated with low ThT fluorescence and high ANS signal (Figure 4). Asn14 is the only H-bond donner/acceptor residue within the 12–17 domain, and removal of Asn amide side chain could interfere with the protofilament interactions necessary for fibril packaging. It was previously reported that the substitution of Asn14 by Ala inhibits IAPP self-assembly (Koo et al., 2008), although this earlier study was performed using the N-truncated IAPP_8-37_ and under different conditions. Replacement of Phe15 by an alanine delayed the appearance of ThT and ANS-positive signals, and β-sheet-rich assemblies. In fact, no fibrillar aggregates could be observed by TEM after 48 h incubation, underlining the importance of this hydrophobic side chain in amyloid formation. Nonetheless, after prolonged incubation time, *i.e.* 168 h, amyloid fibrils could be observed by TEM for the F15A derivative (Supplementary Figure S8). The contributions of Phe15 in IAPP amyloidogenesis remains somewhat ambiguous. In fact, while the replacement of Phe15 by Ala, or Ser, accelerated the aggregation of the IAPP_8-37_ fragment (Wiltzius et al., 2009), the F15A substitution within full-length IAPP abrogated its self-assembly (Bakou et al., 2017), as observed herein. Moreover, using short heptapeptides corresponding to the 12-18 region of IAPP, Phe side chain was shown to delay significantly amyloid self-assembly, while not being fully required for fibrillogenesis (Milardi et al., 2011). Besides, substituting Phe15 by the non-aromatic hydrophobic residue Leu, was shown to accelerate amyloid formation of full-length IAPP under conditions closely related to those used in the present study (Tu and Raleigh, 2013), suggesting the importance of hydrophobicity within this segment. As observed for unmodified IAPP, a random coil-to-β-sheet conformational conversion was observed after 24 h incubation for L16A derivative, which was associated with a ThT-positive signal and the presence of fibrils. These observations are somewhat contradictory with a previous study showing that the L16A substitution reduces substantially IAPP self-assembly, albeit these earlier results were obtained in presence of 2% HFIP, which is known to markedly alter IAPP self-recognition (Bakou et al., 2017). Substituting Val17 by Ala delayed the conformational transition and led to the formation of short and poorly defined fibrils with low ThT and ANS binding properties (Figure 4). Overall, results of Ala-scan revealed that the hydrophobic side chains of residues Leu12, Phe15 and Val17 are important for the formation of β-sheet-rich fibrils and further emphasized the importance of conducting such studies with full-length IAPP as peptide fragments are not recapitulating the sequence effect.

### Side chains of the 12–17 segment modulate lipid membrane perturbation and contribute to IAPP cytotoxicity

**FIGURE 5:**
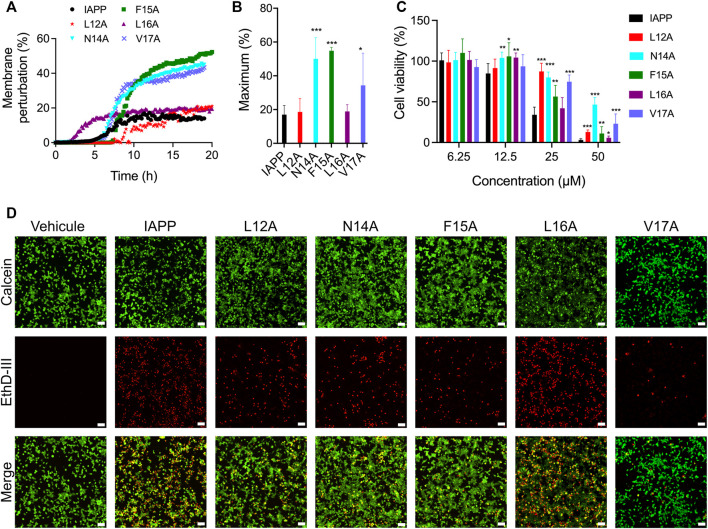
Lipid vesicle perturbation and cytotoxicity of Ala-substituted IAPP derivatives. **(A)** Kinetics of lipid membrane perturbation. 500 μΜ of calcein-loaded LUVs (DOPC:DOPG; 9:1) were exposed to 25 μM peptide in 20 mM Tris-HCl, 150 mM NaCl, pH 7.4, and fluorescence was measured every 10 min. **(B)** Maximum lipid membrane perturbation of at least four assays performed with different peptide lots. Data are expressed as the mean ± S.D. **(C)** INS-1E cells were treated with freshly dissolved peptides for 24 h and metabolic activity was measured by means of resazurin reduction. Data of at least five assays were averaged and expressed as the mean ± S.D. **(D)** Representative fluorescence microscopy images showing the distribution of live (green) and dead (red) INS-1E cells after treatment with 25 μΜ peptides for 24 h. Scale bar: 50 μm. **(B,C)** Statistically significant difference (between IAPP and analogs) was established at (*) *p* < 0.05; (**) *p* < 0.005; (***) *p* < 0.0005.

Considering that the side chains of the 12-17 region are actively participating in IAPP amyloid formation, the impact of the X-to-Ala substitution on membrane perturbation and cytotoxicity was investigated. Whereas multiple studies have indicated that prefibrillar oligomers are the most toxic proteospecies (Abedini et al., 2016; Raleigh et al., 2017), the underlying mechanisms are still the matter of active debates. Among these cellular mechanisms, which include apoptosis, oxidative stress, mitochondrial dysfunction, and endoplasmic reticulum stress (Li et al., 2009; Gurlo et al., 2010; Magzoub and Miranker, 2012; Cadavez et al., 2014; Park et al., 2014), the perturbation of the plasma membrane remains one of the most studied hypotheses. Perturbation of the cell plasma membrane lipid bilayer by the oligomers, and/or by the process of amyloid self-assembly, has been associated with pore-like formation, carpet-like mechanisms, or the recently proposed lipid-chaperone hypothesis (Brender et al., 2012; Sciacca et al., 2020). The ability of the Ala-substituted derivatives to induce leakage of lipid bilayer was evaluated using synthetic anionic LUVs composed of DOPC and DOPG at molar ratio of 9:1 (DOPC:DOPG). This molar ratio between zwitterionic (DOPC) and anionic (DOPG) lipids is within the range of what has been reported for β-pancreatic plasma membrane, which is composed of between 2.5 and 13% anionic lipids (Sasahara, 2018). LUVs loaded with calcein were incubated in presence of 25 μM peptide and the fluorescence was measured every 10 min. For unmodified IAPP, an increase of calcein fluorescence associated with membrane leakage was observed after 5 h incubation and a fluorescence of 17% of that obtained in presence of Triton X-100 was reached after 10 h (Figures 5A,B). Interestingly, successive substitution of Asn14, Phe15 and Val17 by Ala increased the capacity of IAPP to perturb anionic LUVs, with maximum fluorescence respectively reaching 50, 55 and 34% (Figures 5A,B). The analogs L12A and L16A showed a similar capacity to the unmodified IAPP to cause calcein release, albeit the L16A substitution accelerated lipid bilayer damage. These observations indicate that, although the sequential replacements of residues Leu12, Phe15 and Val17 by Ala delayed aggregation in homogeneous aqueous solution (Figures 3, 4), these substitutions did not reduce the capacity of the peptide to permeabilize anionic LUVs.

As LUVs do not recapitulate the complexity of cell plasma membrane (Zhang et al., 2017; Quittot et al., 2021), and numerous mechanisms are implicated in IAPP cytotoxicity, we evaluated the capacity of Ala-substituted analogs to affect viability of the pancreatic-derived cells. INS-1E cells were treated with freshly dissolved peptides and incubated for 24 h before measuring viability with resazurin reduction. IAPP induced a concentration-dependent decrease of metabolic activity, with less than 10% of viability observed at 50 μM (Figure 5C). While the L16A derivative showed a comparable cytotoxicity to the unmodified peptide, successive replacement of Leu12, Asn14, Phe15 and Val17 by Ala reduced IAPP toxicity. Particularly, the amide side chain of Asn-14 appears to play a key role, with 50% of cellular viability measured at 50 μM. Results obtained with resazurin reduction, which is mainly associated with mitochondrial oxidoreductase activity (Magnani and Bettini, 2000), were corroborated using a viability assay. As observed by fluorescence microscopy, 24 h treatment with 25 μM freshly solubilized IAPP induced a sharp increase in the number of red cells and a decrease of green cells (Figure 5D). The green fluorescence is associated with intracellular esterase activity, while the red fluorescence is linked to the loss of plasma membrane integrity. Concurring with the resazurin-based assay, the L12A, N14A, F15A and V17A analogs exhibited a decreased toxicity compared to unmodified IAPP, while the L16A peptide caused similar extent of cell death. These results indicate that side chains of the 12–17 domain are implicated in IAPP-mediated cytotoxicity towards pancreatic β-cells, with Leu16 isobutyl side chain playing a limited role. No correlation between the capacity of the peptides to permeabilize anionic LUVs with their toxicity was observed, highlighting that IAPP-mediated cell death is multifaceted.

### Destabilizing the local conformation by Pro incorporation modulates IAPP self-assembly

**FIGURE 6:**
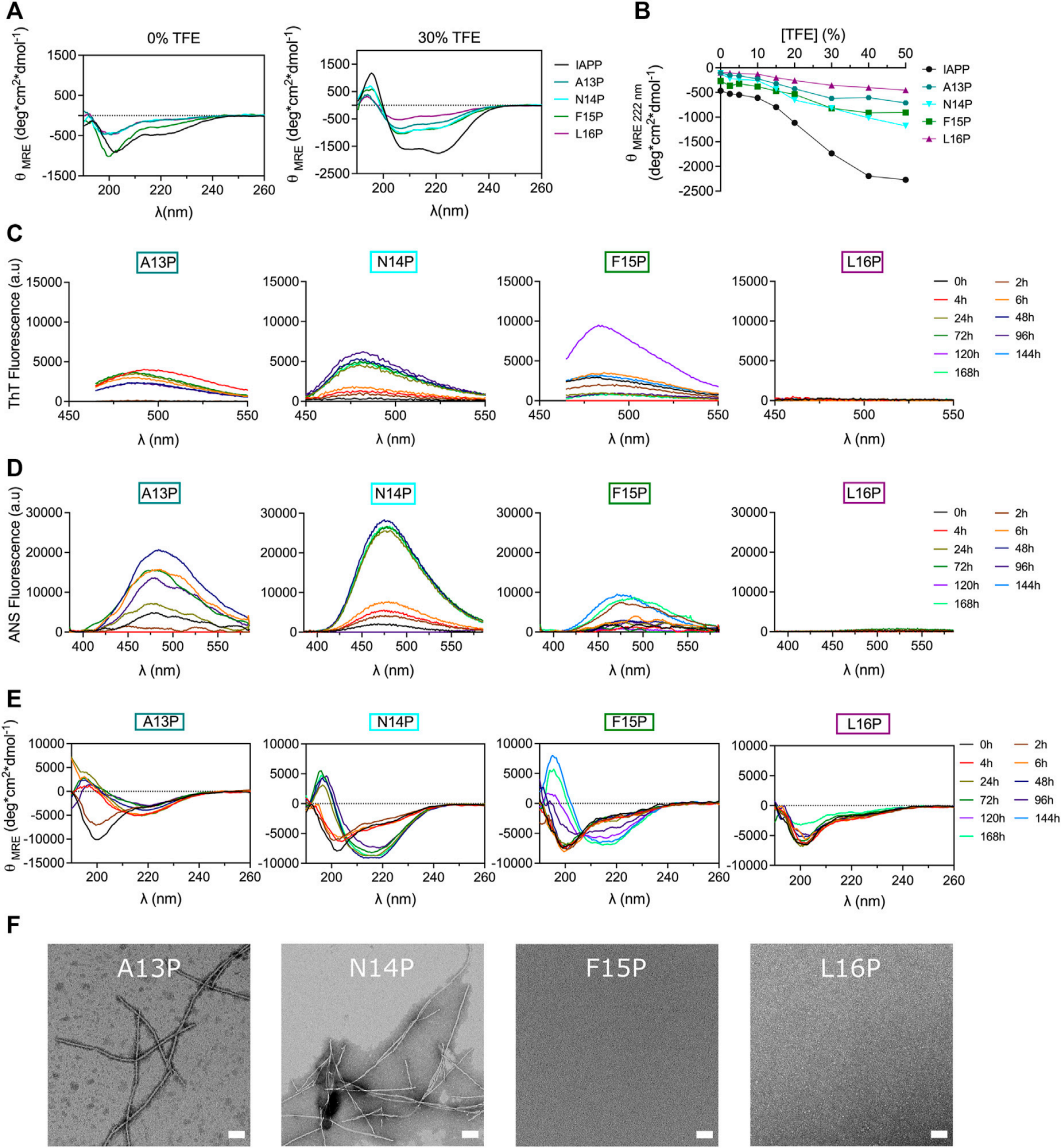
Effects of Pro substitution on IAPP amyloid self-assembly. **(A)** Far-UV CD spectra of peptides in Tris-HCl 20 mM in presence, or absence, of 30% TFE. **(B)** Effect of TFE on peptide ellipticity measured at 222 nm. **(C–E)** Peptides were incubated under quiescent conditions in 20 mM Tris-HCl buffer, pH 7.4, at a concentration of 50 μM before being diluted at 25 μM for **(C)** ThT fluorescence, **(D)** ANS fluorescence and **(E)** CD analysis. **(F)** TEM images obtained after for 48 h incubation. Scale bar: 100 nm.

Different models have been proposed regarding the intermediates populating the lag phase and/or initiating amyloid formation, including the β-hairpin model (Dupuis et al., 2011), the parallel β-sheet model (Buchanan et al., 2013; Maj et al., 2018), and the helical intermediate model (Abedini and Raleigh, 2009; Hebda and Miranker, 2009; De Carufel et al., 2015). The latter advocates that IAPP self-recognition is thermodynamically linked with secondary helical transition within the 5–20 segment, as observed in coiled-coil motif formation, ultimately aligning the C-terminal region and triggering the formation of intermolecular β-sheets (Wiltzius et al., 2009). Molecular dynamics simulations revealed that IAPP oligomerization is promoted by helix-helix association involving hydrophobic side chains of the 12-17 region (Laghaei et al., 2011). The potential implication of helical folding in IAPP amyloid formation was probed by incorporating residues within the core of the 12–17 segment, *i.e.* at positions 13 to 16, to modulate the local secondary conformational ensemble and/or backbone flexibility, such as Pro, Gly and Aib. First, residues Ala13 to Leu16 were successively substituted by Pro, which is well known to destabilize helical folding (Li et al., 1996) and to inhibit amyloid formation (Moriarty and Raleigh, 1999) owing to its conformational rigidity. Titration with the helical-inducing solvent trifluoroethanol (TFE) by far-UV CD spectroscopy confirmed that consecutive proline incorporation destabilizes IAPP helical folding (Figures 6A,B).

Next, Pro-substituted peptides were incubated at 50 μM under fully quiescent conditions and the solutions were periodically evaluated. Interestingly, substitution of Ala13 by Pro accelerated amyloid formation, as observed with the random coil→β-sheet transition detected after 4 h and with the ThT and ANS positive signal arising after 6 h incubation (Figure 6). Prototypical fibrils were observed by TEM, albeit some small aggregates were also detected (Figure 6F). In comparison to their Ala-substituted counterpart, incorporation of Pro at position Asn14 and Phe15 accelerated IAPP conformational transition. For instance, N14P showed a β-sheet CD signal associated with ThT and ANS fluorescence after 24 h incubation (Figure 6), whereas N14A needed 48 h to undergo this conformational transition (Figure 4). In fact, AFM images revealed a dense network of fibrils for the N14P peptide after 24 h incubation, while only poorly defined fibrils were observed for the analog N14A (Supplementary Figure S9). Similarly, F15P derivative formed typical fibrils after 96 h incubation, whereas only small aggregates were observed for the F15A analog (Supplementary Figure S10), in agreement with ThT and ANS fluorescence and CD analysis (Figure 6). It is worth mentioning that the Phe15-to-Ala substitution still delayed the formation of amyloid fibrils, as observed with the absence fibrillar aggregates upon 48 h incubation of the F15P analogs (Figure 6F). These findings regarding analogs A13P, N14P and F15P are intriguing since the incorporation of a Pro residue constitutes a well-known strategy to inhibit amyloid aggregation (Abedini and Raleigh, 2006; Abedini et al., 2007; Kraus, 2016; Ridgway et al., 2020). In sharp contrast, substitution of Leu16 by Pro completely inhibited amyloid formation, as noticed with the absence of a ThT and ANS signal and the random coil conformation observed after 7 days incubation (Figure 6). The inhibitory effect of the L16P substitution is not associated with the absence of the hydrophobic isobutyl side chain, as L16A showed similar self-assembly propensity to unmodified IAPP (Figure 4), indicating that the conformational rigidity imposed by the pyrrolidine loop is likely the cause. This result concurs with a previous study showing that the H18P substitution into full-length IAPP prevents amyloid formation (Akter et al., 2018). Overall, destabilizing the α-helix by successively incorporating a Pro residue at positions Ala13 to Phe15 accelerates, in comparison to Ala-modified counterparts, the process of self-assembly, whereas the opposite effect was observed at position Leu16, indicating that the effect of X-to-Pro substitution on IAPP self-assembly is site-specific. This observation suggests that destabilizing helical folding within the 13–15 segment promotes IAPP self-assembly by facilitating the secondary conformational conversion from α-helical oligomeric intermediates into β-sheet quaternary species, *en route* to amyloid formation. In contrast, the inhibitory effect of the Leu16-to-Pro substitution proposes that a helical conformation within the vicinity of position 16 could constitute an obligatory on-pathway oligomeric intermediates to fibril formation.

### Glycine substitution site-specifically affects IAPP self-assembly

**FIGURE 7:**
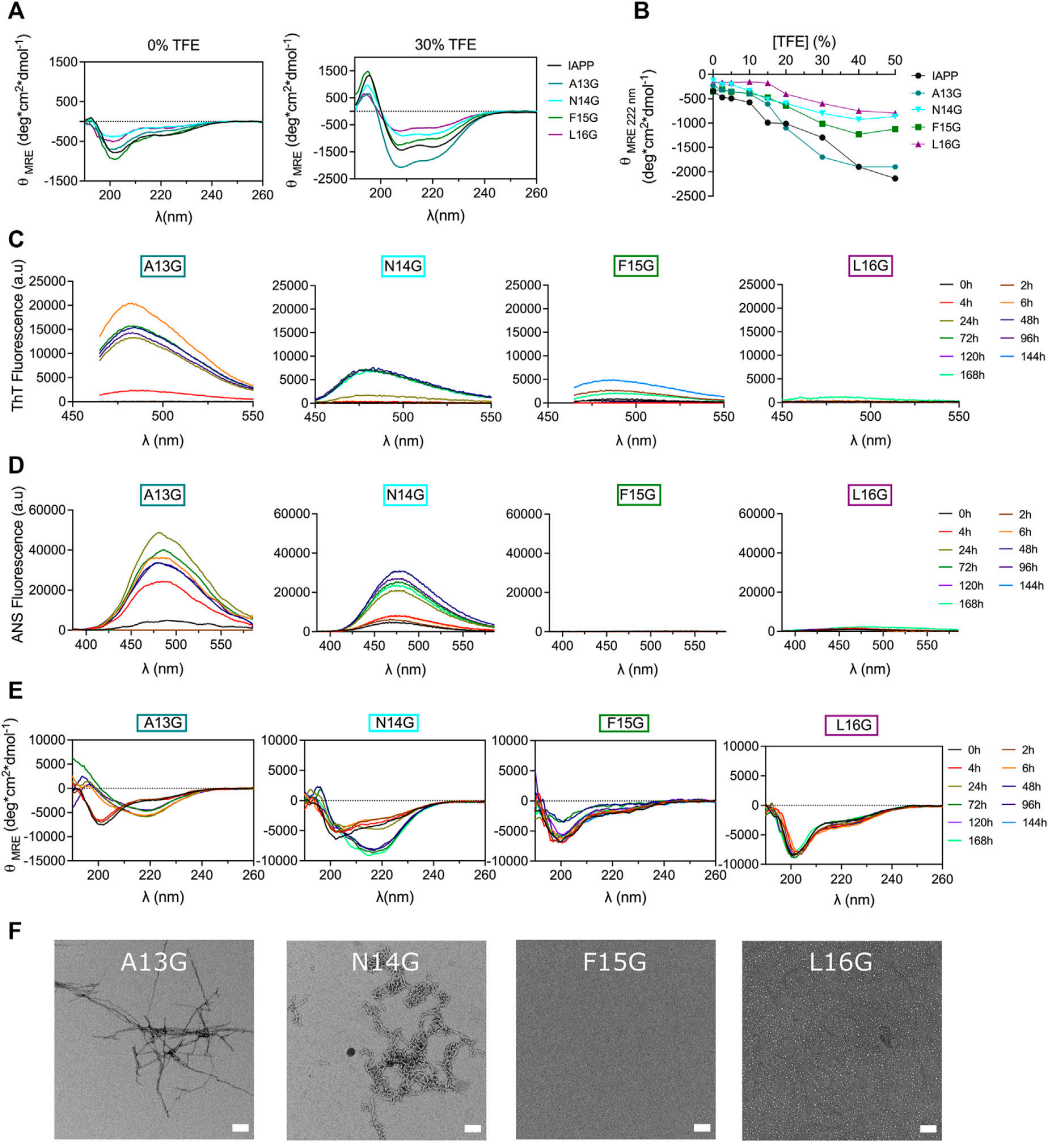
Effects of Gly substitution on IAPP amyloid self-assembly. **(A)** Far-UV CD spectra of IAPP and Gly-substituted analogs in Tris-HCl 20 mM in presence, or absence, of 30% TFE. **(B)** Effect of TFE on the mean residue ellipticity measured at 222 nm. **(C–E)** Peptides were incubated under quiescent conditions in 20 mM Tris-HCl buffer, pH 7.4, at a concentration of 50 μM before **(C)** ThT fluorescence, **(D)** ANS fluorescence and **(E)** CD analysis. **(F)** TEM images obtained after for 48 h incubation. Scale bar: 100 nm.

Glycine residue, bearing a single hydrogen atom as side chain, was successively introduced into the core of the 12–17 domain to further probe the effect of conformational modulation on amyloid formation. In contrast to Pro, which increases local conformational rigidity, Gly enhances peptide backbone flexibility (Nick Pace and Martin Scholtz, 1998). As revealed by TFE titration, consecutive incorporation of Gly at positions Asn14, Phe15 and Leu16 destabilizes the propensity of IAPP to adopt an α-helix, whereas the A13G analog showed similar propensity to helical folding to the unmodified peptide (Figures 7A,B). These observations concur with the fact that glycine can act as a «helix breaker » or a «helix inductor», depending on the local sequence (Li and Deber, 1992). Monomerized Gly-substituted derivatives were incubated in 20 mM Tris-HCl, pH 7.4 at room temperature without agitation at 50 μΜ. CD spectroscopy revealed that the A13G analog was subjected to a conformational transition within 6 h incubation, which was associated with an increase of ThT and ANS fluorescence (Figure 7), suggesting that the substitution of Ala13 by a Gly accelerates IAPP amyloid self-assembly. TEM images of A13G confirmed the presence of fibrillar aggregates (Figure 7F). Substitution of Asn14 by Gly delayed the random coil-to-β-sheet shift and the increase of ThT and ANS fluorescence, in comparison to the unmodified IAPP and the N14A analog. Moreover, N14G fibrils obtained after 48 h incubation were short, less defined, and twisted (Figure 7F), compared to the ones obtained with the Ala-substituted counterpart. Successive incorporation of a Gly at positions Phe15 and Leu16 delayed IAPP amyloid formation, as observed by CD spectroscopy, ThT and ANS fluorescence and TEM imaging (Figure 7). Oligomer-like structures were observed by TEM after 48 h incubation of the L16G derivative, whereas no aggregates could be detected for F15G after 48 h. Nonetheless, some partially dispersed amyloid fibrils could be observed by TEM after 7 days incubation (Supplementary Figure S12). These observations highlight inequivalence in site-specific Gly substitution; increasing the peptide backbone flexibly at position Ala13 promotes IAPP self-assembly, while amyloid formation is inhibited when Gly is successively incorporated at positions 15 and 16.

### Successive incorporation of aminoisobutyric acid at positions Phe15 and Leu16 inhibits amyloid formation

**FIGURE 8:**
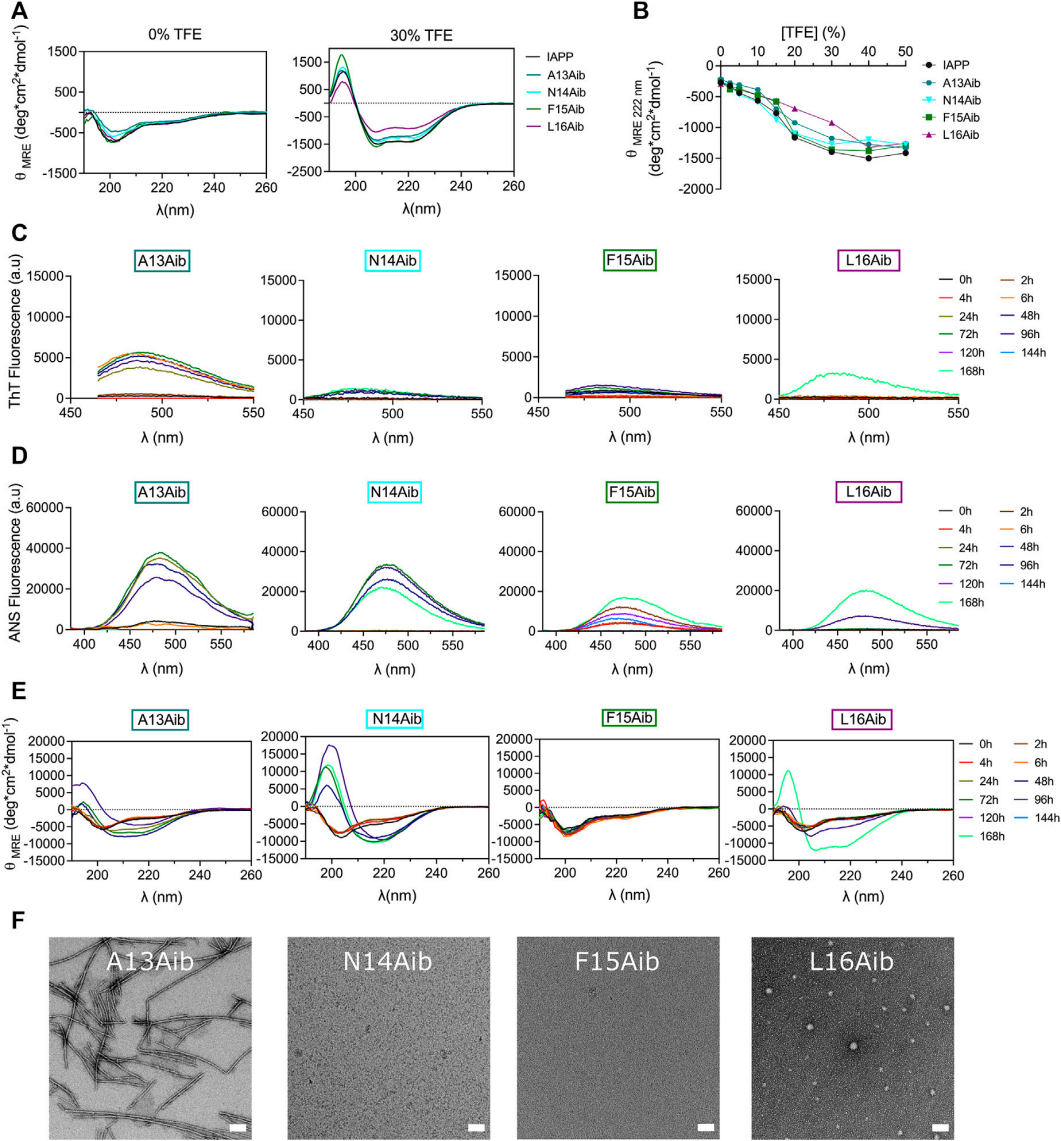
Effects of backbone α-methylation on IAPP amyloid self-assembly. **(A)** Far-UV CD spectra of peptides in Tris-HCl 20 mM in presence, or absence, of 30% TFE. **(B)** Effect of TFE on the mean residue ellipticity measured at 222 nm. **(C–E)** Peptides were incubated under quiescent conditions in 20 mM Tris-HCl buffer, pH 7.4, at a concentration of 50 μM before being diluted at 25 μM for **(C)** ThT fluorescence, **(D)** ANS fluorescence and **(E)** CD analysis. **(F)** TEM images obtained after for 48 h incubation. Scale bar: 100 nm.

The aminoisobutyric acid (Aib), which corresponds to an additional methyl group on the α-carbon of alanine (α-methylalanine) was successively introduced within the 13–16 segment to affect the local secondary conformation. Aib is known to promote helical folding through the Thorpe-Ingold effect (Karle, 2001; Toniolo et al., 2001). However, no clear effect on the secondary structure of IAPP in aqueous buffer could be observed upon Aib substitution (Figure 8A) and Aib-based analogs displayed similar TFE-induced helical folding to IAPP (Figure 8B). Interestingly, α-methylation of Ala13 led to the formation of well-defined ThT-positive fibrils upon 48 h incubation, which were associated with a CD spectrum characterized by a mixture of α-helix and β-sheet (Figure 8). This CD spectrum evolved into a typical β-sheet-rich CD signal after 7 days (Figure 8E). In contrast, replacement of Asn14 by Aib delayed dramatically amyloid formation, with the quasi absence of ThT signal, the appearance of a β-sheet CD signal after 72 h incubation and no visible fibrils observable by TEM. Considering that the N14A analog readily assembled into fibrillar aggregates (Figure 4), the low amyloidogenicity of the N14Aib derivative is not associated with the removal of the amide side chain, but likely resides in the backbone steric hindrance caused by the α-methylation. The F15Aib analog remained in a random coil conformation after 7 days, with low ThT fluorescence, indicative of a complete inhibition of fibrillization, as confirmed by TEM (Figure 8, Supplementary Figure S13). Similarly, substitution of Leu16 by an Aib severely delayed the conformational transition, with the presence of a CD signal with two minima at 208 and 222 nm after 7 days incubation. Interestingly, this emergence of a helical CD spectrum correlates with an increase of ThT and ANS fluorescence. Small dot-like nanostructures, closely related to oligomers, were observed for L16Aib by TEM after 48 h incubation. Considering that the L16A peptide readily forms amyloid fibrils (Figure 4), these results indicate that the decrease of amyloidogenecity caused by the Leu-to-Aib replacement is not associated with the absence of the hydrophobic isobutyl chain. Overall, backbone α-methylation reveals that position 13 tolerates the imposed steric hindrance, whereas incorporation of Aib at positions 15 and 16 inhibits amyloid formation, suggesting that the site-specific incorporation of Aib could be a strategy to inhibit amyloid aggregation of IAPP peptide drugs.

### Modulating the local conformational flexibility of the 13–16 segment alters cytotoxicity

**FIGURE 9:**
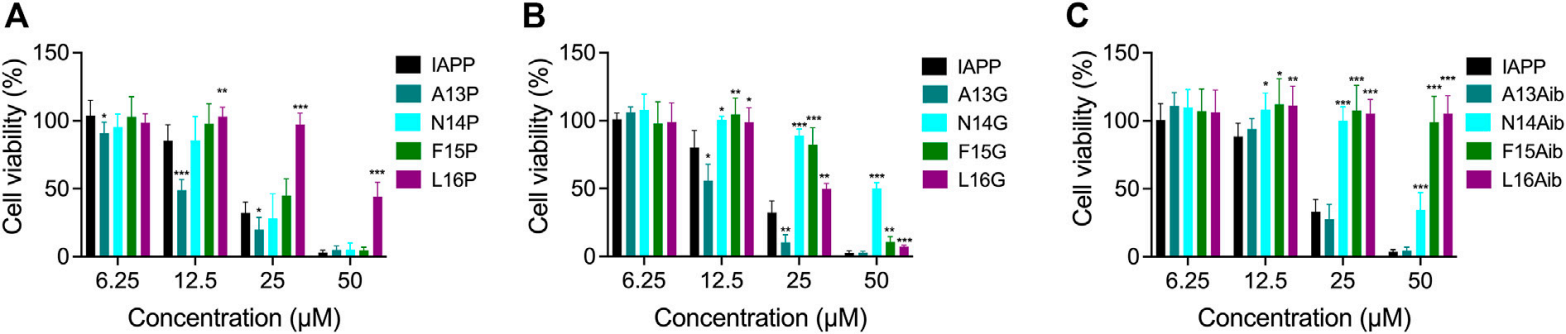
Cytotoxicity of Pro, Gly and Aib substituted IAPP. **(A–C)** INS-1E cells were incubated with increasing concentrations of freshly dissolved peptides for 24 h. Cell viability was measured using the resazurin reduction assay and compared with cells treated with vehicle. Data of at least five assays were averaged and expressed as the mean ± S.D. Results were analyzed using Student’s t test, and statistically significant difference between IAPP and analogs was established at: (*) *p* < 0.05; (**) *p* < 0.005; (***) *p* < 0.0005.

After observing that residues modulating the local secondary structure and/or the backbone flexibility affect IAPP amyloidogenicity, the impact of these substitutions on cytotoxicity was evaluated. Pancreatic INS-1E cells were treated with freshly solubilized peptides and incubated for 24 h before measurement of metabolic activity by resazurin reduction. Whereas substitution of Ala13 by Pro increased the cytotoxicity of IAPP at 12.5 and 25 μM, the L16P derivative was found to be less cytotoxic than unmodified IAPP, particularly at 25 and 50 μM (Figure 9A). N14P and F15P showed similar concentration-dependant toxicity to unmodified IAPP. As observed for A13P, A13G showed a higher cytotoxicity compared to unmodified IAPP (Figure 9B), suggesting that the destabilization of α-helix conformation within this segment promotes the formation of toxic intermediates. Oppositely, consecutive incorporation of a Gly at positions Asn14, Phe15 and Leu16 significantly reduced IAPP cytotoxicity (Figure 9B). Especially, the N14G peptide was poorly toxic for pancreatic cells, even at 50 μM, although this analog was prone to amyloid aggregation (Figure 7). This observation indicates that not all oligomers are cytotoxic, as shown with rIAPP (Abedini et al., 2016), and that specific structural motifs modulate toxicity (Godin et al., 2019). Effect of Aib substitution on toxicity was site-specific, with A13Aib derivative showing similar toxicity to the unmodified IAPP and analogs N14Aib, F15Aib and L16Aib being poorly cytotoxic (Figure 9C). Notably, F15Aib and L16Aib analogs, which were incompetent to assemble into amyloid aggregates (Figure 8), were also not toxic at the highest concentration tested, indicative of some correlation between amyloidogenicity and toxicity. Considering the toxicity of IAPP and Ala-modified analogs (Figure 5), these results indicate that the IAPP-mediated cytotoxicity tolerates alterations of backbone flexibility at position 13, whereas backbone conformational restriction at position Leu16 hinders the formation of culprit proteospecies.

## Conclusion

The present investigation underlines the importance of the 12-17 central region of IAPP for amyloid aggregation and associated cell toxicity, a segment which has been historically unrecognized in comparison to the 20–29 domain. Molecular dynamics simulation and *in silico* prediction of amyloidogenicity suggested that the 12–17 domain of IAPP participates in self-recognition and is most likely implicated in the initial steps of oligomerization. Ala scanning revealed that the hydrophobic side chains of Leu12, Phe15 and Val17 are particularly important to drive the self-assembly process, while these residues are not mandatory for IAPP to permeabilize anionic LUVs and to reduce the viability of pancreatic β-cells. Moreover, while the amide side chain of Asn14 is not critical for IAPP amyloidogenicity, it is contributing to the cytotoxic potential of the peptide. Interestingly, the destabilization of the α-helix secondary structure by Pro incorporation hastened amyloid formation when the pyrrolidine ring was introduced at positions Ala13, Asn14 and Phe15, in comparison to their respective Ala-substituted counterparts, suggesting that an α-helix involving the 13–15 segment does not constitute an obligatory *on-pathway* quaternary intermediate, as previously proposed (De Carufel et al., 2015). In sharp contrast, the destabilization of helical folding with the Leu16-to-Pro substitution inhibited amyloid formation, suggesting that a helical conformation involving position 16 could constitute an obligatory on-pathway oligomeric intermediates to fibril formation. Whereas the Leu16 isobutyl chain is not critical for IAPP self-assembly and β-cell toxicity, modulating the peptide backbone flexibility at position 16 inhibited amyloid formation and reduced cytotoxicity. In fact, incorporation of a methyl group on the α-carbon of Phe15 and Leu16, *i.e.* F15Aib and L16Aib, abrogated IAPP fibrillization and toxicity, suggesting that Aib incorporation constitutes a promising strategy to convert IAPP from highly aggregation prone peptide to a non-amyloidogenic peptide. It will now be interesting to evaluate if the non-amyloidogenic F15Aib and L16Aib derivatives can activate the AMY receptors and to test their ability to inhibit amyloid formation of unmodified IAPP.

## Data Availability

The raw data supporting the conclusion of this article will be made available by the authors, without undue reservation.
