# Supplementary material for: Contribution of the 12–17 hydrophobic region of islet amyloid polypeptide in self-assembly and cytotoxicity
Source: Front Mol Biosci. 2022 Oct 3;9:1017336. doi: 10.3389/fmolb.2022.1017336 (PMC9573943; doi:10.3389/fmolb.2022.1017336)
Supplement: Supplementary file 1 [file DataSheet1.PDF]

Supporting Information

Contribution of the 12-17 Hydrophobic Region of Islet Amyloid Polypeptide in Self-Assembly and Cytotoxicity

Mathilde Fortier[1,2], Mélanie Côté-Cyr[1,2], Vy Nguyen[1,2], Margaryta Babych[1,2], Phuong Trang Nguyen[1,2], Roger Gaudreault[1,3,] and Steve Bourgault[1,2,]**

[1]Department of Chemistry, Université du Québec à Montréal, C.P. 8888, Succursale Centre-Ville, Montreal, H3C 3P8, Canada
[2]Quebec Network for Research on Protein Function, Engineering and Applications, PROTEO, Canada
[3]Department of Physics, Université de Montréal, C.P. 6128, Succursale Centre-ville, Montreal (QC), H3C 3J7 Canada

***Correspondence:**
Professor Steve Bourgault
Department of Chemistry
Université du Québec à Montréal
C.P. 8888, Succursale Centre-Ville
Montreal (Quebec)
H3C 3P8, Canada
1-514-987-3000 (5161)
bourgault.steve@uqam.ca

Dr. Roger Gaudreault
Department of Physics
Université de Montréal
Campus MIL, B-4047
514-343-7357
roger.gaudreault@umontreal.ca

Table of Content

Table S1. Mass spectrometry analysis of peptides………………………………….... 3

Figure S1. ThT and ANS fluorescence of $IAPP_{12-17}$………………………………… 4

Figure S2. Convergence of IAPP homodimer over the MD trajectory……………… 5

Figure S3. Secondary structure of IAPP homodimer……………………………….. 6

Figure S4. Interchain pairwise (residue-residue) minimum distance………………... 7

Figure S5. Residue-pairwise intrachain distance maps of IAPP discrete monomers.. 8

Figure S6. Number of interchain pairwise (residue-residue) contacts……………. 9

Figure S7. In silico analysis of alanine substitution within the 12-17 domain……… 10

Figure S8. TEM image of F15A IAPP after 168h incubation……………………….. 11

Figure S9. Effect of substitutions at position Asn14 on amyloid formation………… 12

Figure S10. Effect of substitutions at position Phe15 on amyloid formation……….. 13

Figure S11. TEM image of F15P IAPP after 168h incubation……………………… 14

Figure S12. TEM image of F15G IAPP after 168h incubation……………………… 15

Figure S13. TEM image of F15Aib IAPP after 168h incubation…………………… 16

Table S1. Mass spectrometry analysis of peptides

Peptide	Theorical molecular weight * (Da)	Experimental molecular weight (Da)
**IAPP**	3903.28	3904.2
**$IAPP_{12\text{-}17}$**	674.83	674.41
**L12A**	3861.20	3860.82
**N14A**	3860.26	3859.88
**F15A**	3827.19	3826.84
**L16A**	3861.20	3860.82
**V17A**	3875.23	3875.25
**A13P**	3929.32	3928.95
**N14P**	3886.29	3885.88
**F15P**	3853.22	3852.88
**L16P**	3887.24	3886.84
**A13G**	3889.26	3888.85
**N14G**	3846.23	3845.88
**F15G**	3813.16	3812.82
**L16G**	3847.18	3844.79
**A13Aib**	3917.40	3916.88
**N14Aib**	3874.28	3873.90
**F15Aib**	3841.21	3840.85
**L16Aib**	3875.23	3874.84

**Theorical molecular weight were obtained with the peptide property calculator (INNOVAGEN, https://pepcalc.com/). Peptides are amidated at their C-terminus and cysteines 2 and 7 form a disulfide bond.*

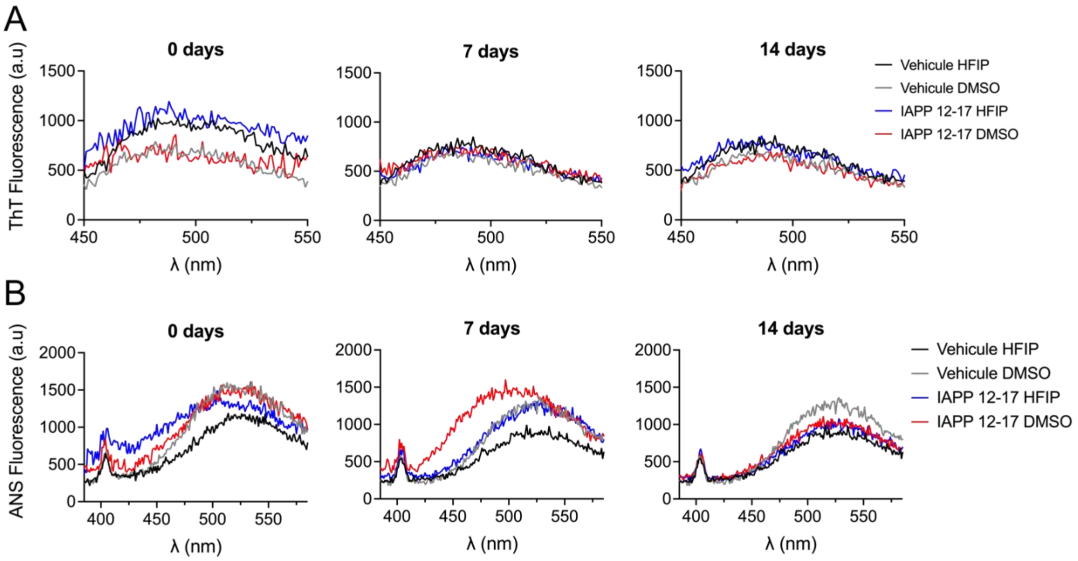

Figure S1. ThT and ANS fluorescence of $IAPP_{12-17}$. At the indicating incubation time, $IAPP_{12-17}$ was diluted to 25 μM in presence of 40 μM ThT, or 25 μM ANS. (A) ThT emission scan after excitation at 440 nm. (B) ANS emission scan after excitation at 355 nm.

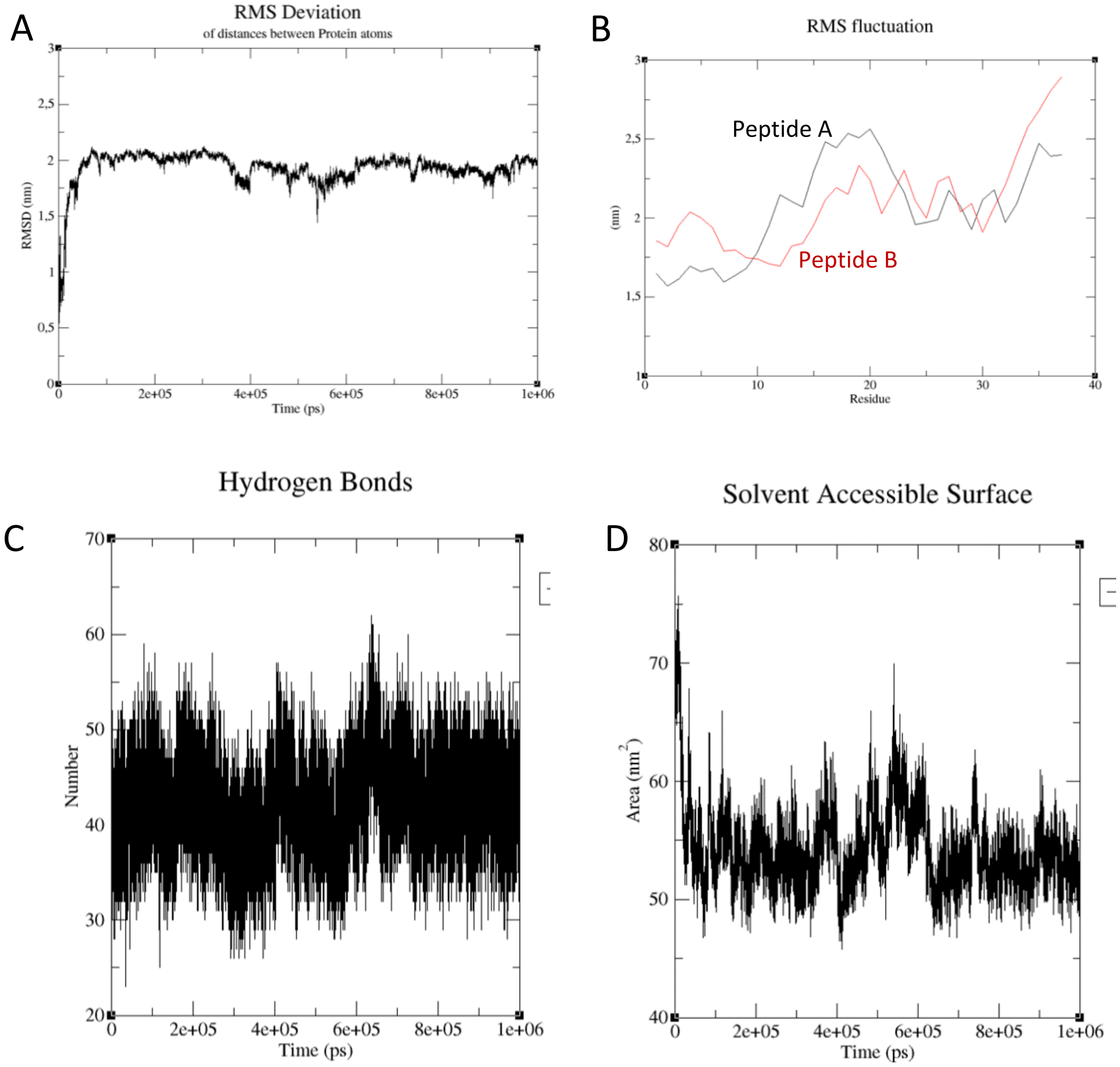

Figure S2. Convergence of IAPP homodimer over the MD trajectory. (A) Root mean square deviation (RMSD) on the backbone atoms (N, C_α, C and O) from peptide structures as a function of time (1000 ns). (B) Root mean square fluctuations (RMSF) of C_α per residue for the 750-1000 ns interval. (C) Number of H-bonds and (D) solvent accessible surface area (SASA) over 1000 ns trajectory.

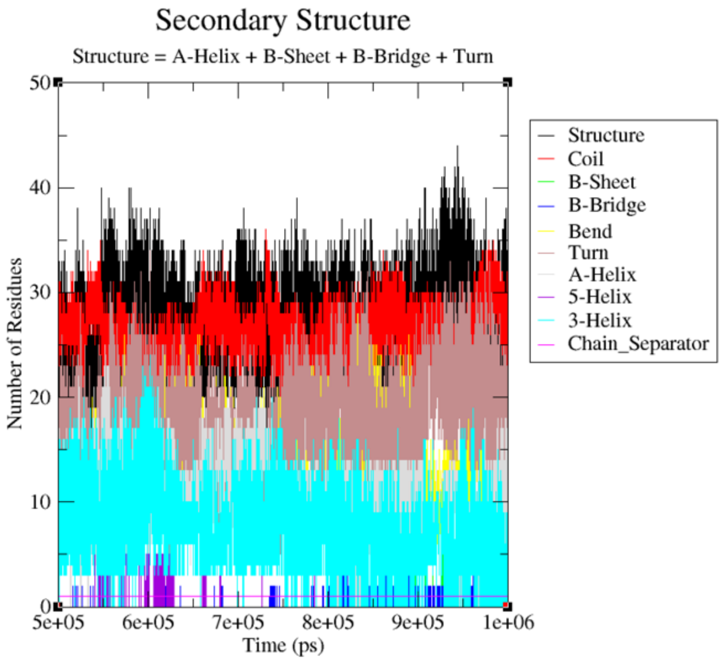

Figure S3: Secondary structure of IAPP homodimer over the 500-1000 ns interval.

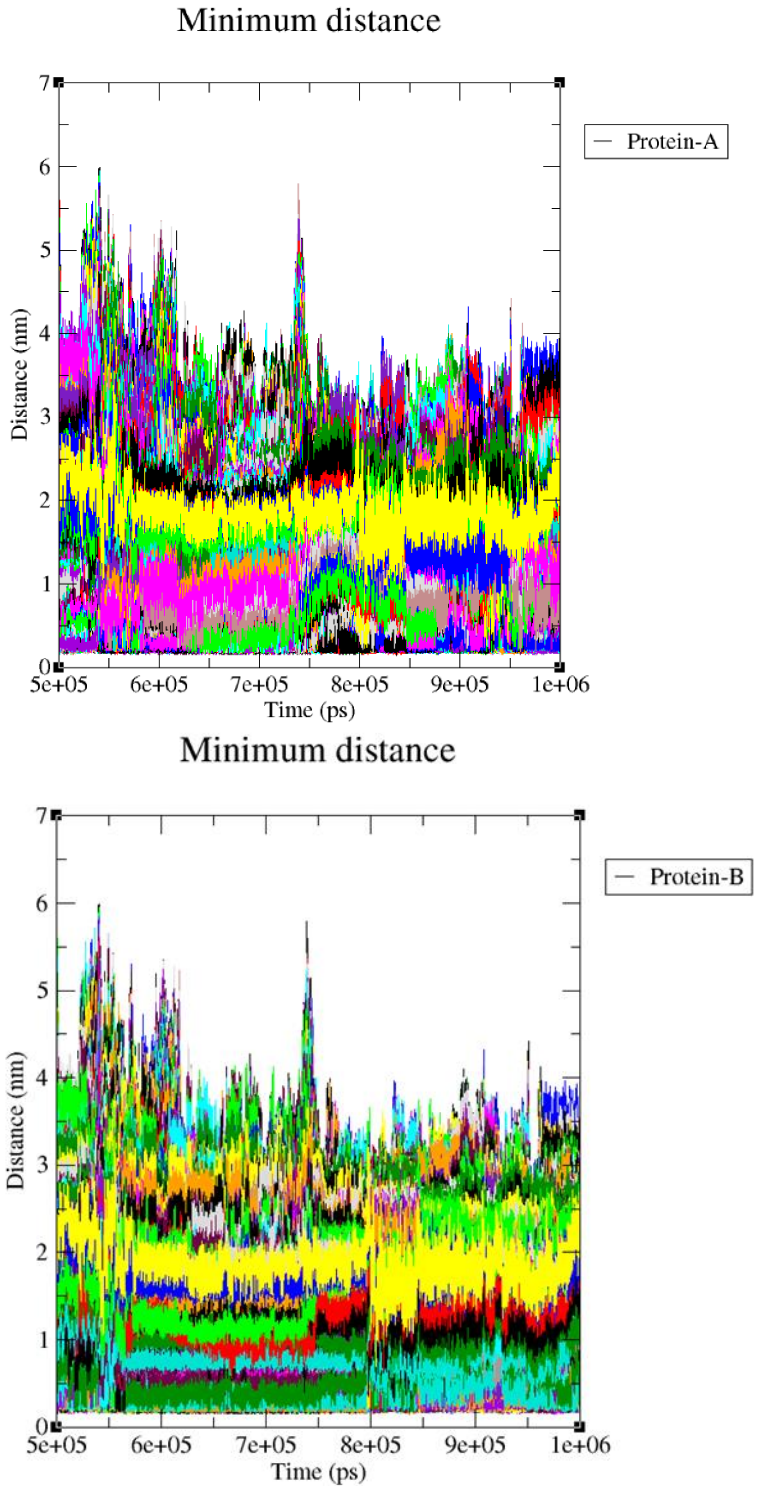

Figure S4: Interchain pairwise (residue-residue) minimum distance over the 500-1000 ns interval for peptide A (top panel) and peptide B (bottom panel).

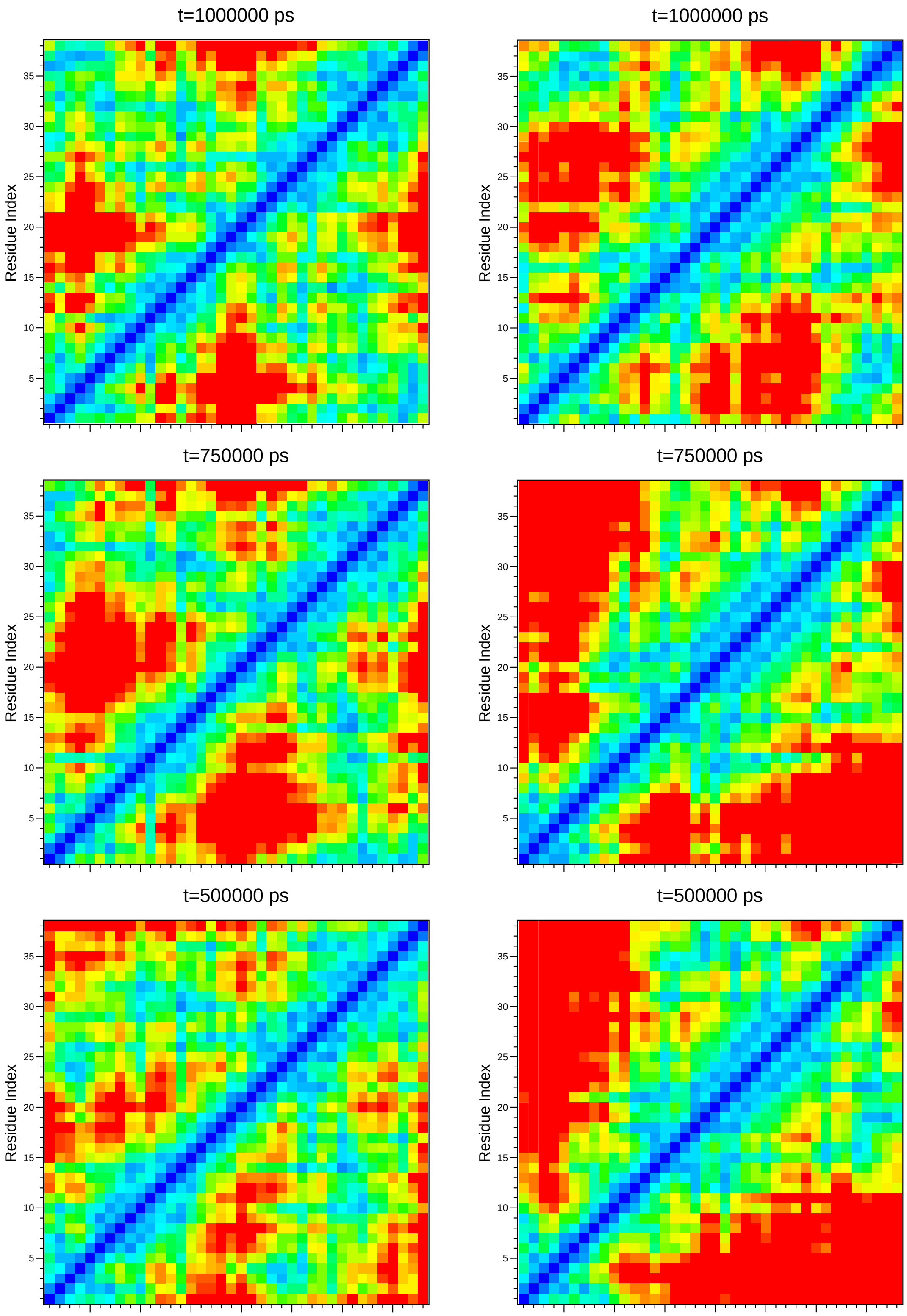

Figure S5: Residue-pairwise intrachain distance maps of IAPP discrete monomers for 500, 750 and 1000 ns for peptide A (left panels) and peptide B (right panels). Highly saturated blue color represents 0 nm whereas red is 1.5 nm distance.

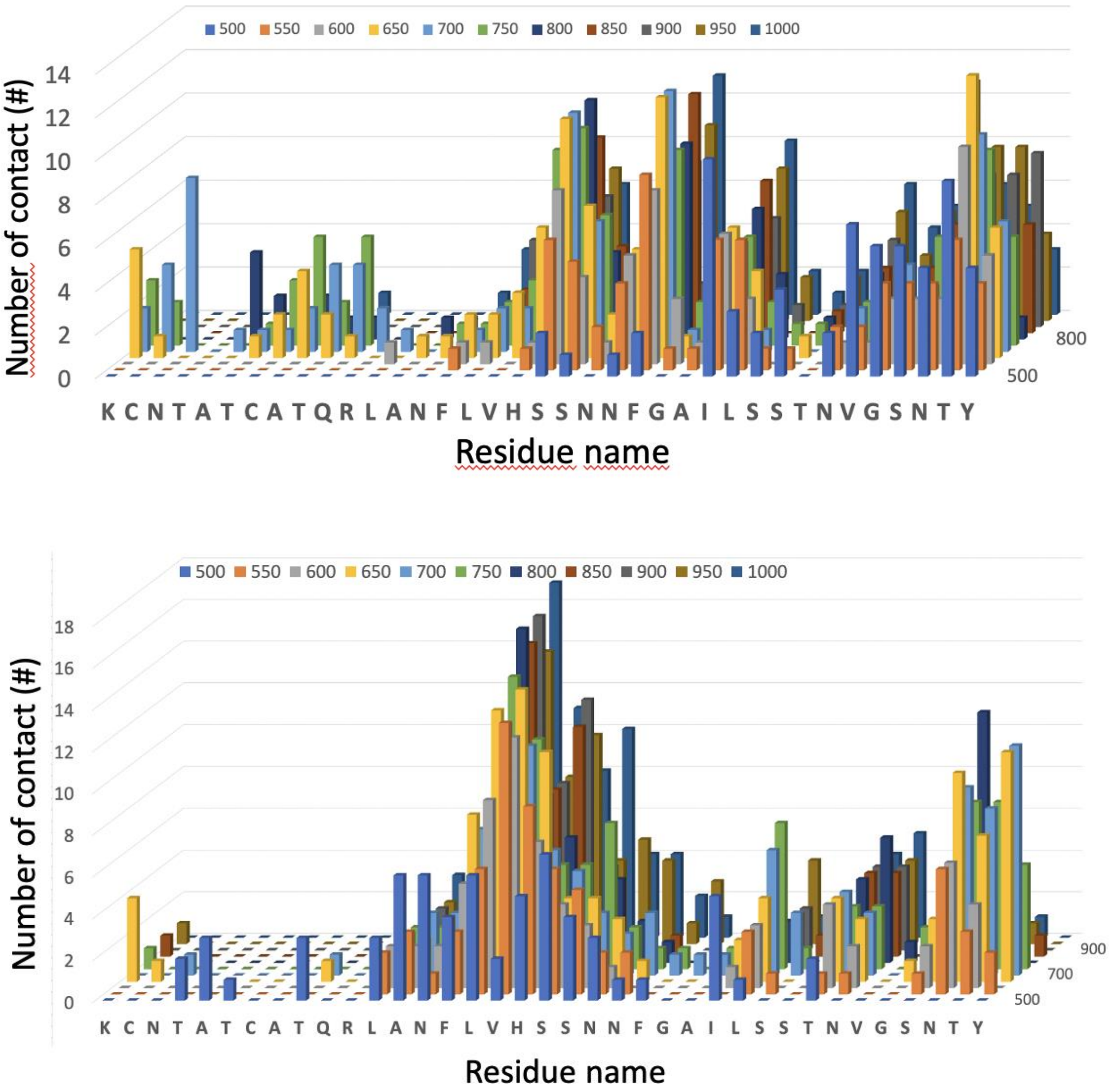

Figure S6: Number of interchain pairwise (residue-residue) contacts (< 0.6 nm distance) for every 50 ns over the 500-1000 ns interval: peptide A (top panel) and peptide B (bottom panel).

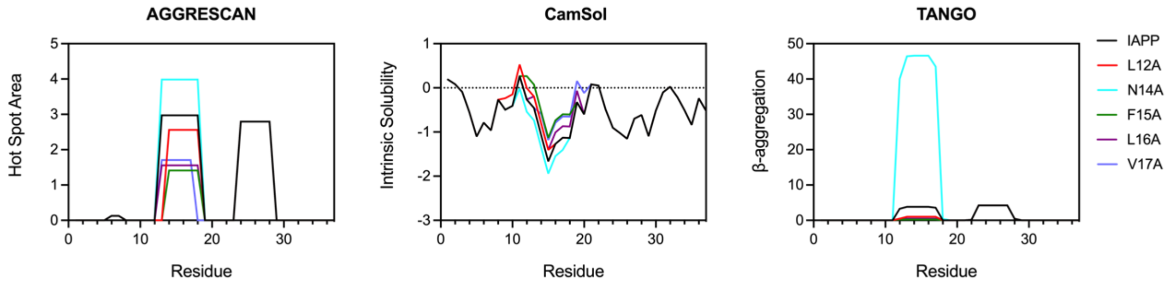

Figure S7. *In silico* analysis of alanine substitution within the 12-17 domain. Analysis was performed with Aggrescan, Camsol and Tango. Predictions were carried out at pH 7.4 and for C-amidated, if possible.

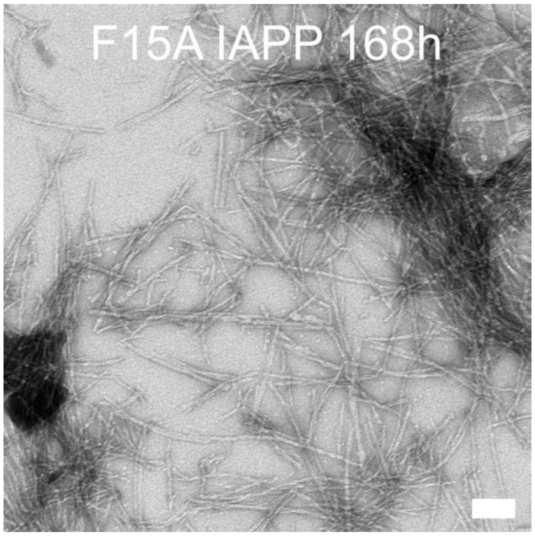

Figure S8. TEM image of F15A IAPP after 168h incubation. Peptide was dissolved at 50 μM in 20 mM Tris-HCl pH 7.4 and incubated at room temperature without agitation for 168 h before TEM analysis. Scale bar: 100 nm.

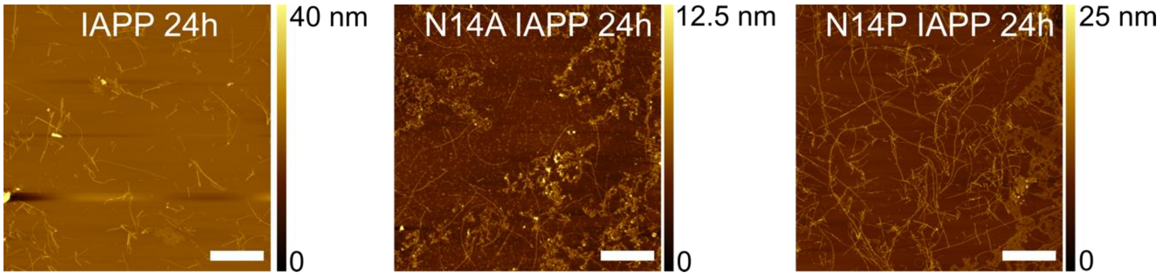

Figure S9. Effect of substitutions at position Asn14 on amyloid formation. Peptides were dissolved at 50 μM in 20 mM Tris-HCl pH 7.4 and incubated at room temperature without agitation for 24 h. Analogs were diluted in 1% acetic acid before being applied on freshly cleaved micas. Scale bar: 2 μm.

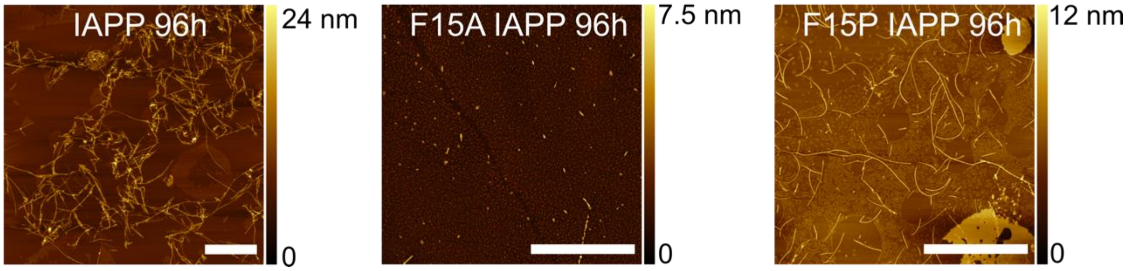

Figure S10. Effect of substitutions at position Phe15 on amyloid formation. Peptides were dissolved at 50 μM in 20 mM Tris-HCl pH 7.4 and incubated at room temperature without agitation for 96 h. Analogs were diluted in 1% acetic acid before being applied on freshly cleaved micas. Scale bar: 2 μm.

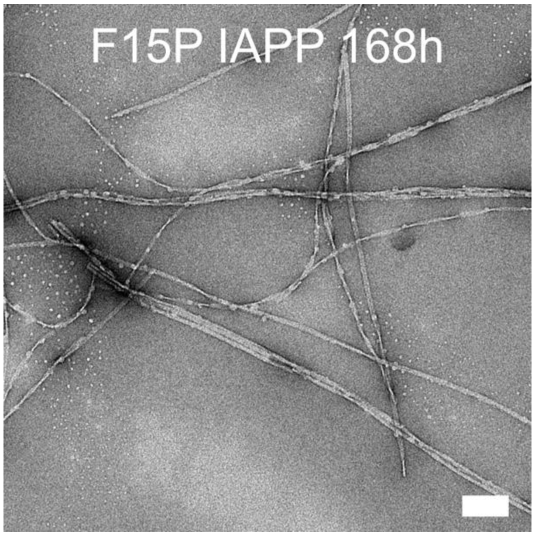

Figure S11. TEM image of F15P IAPP after 168h incubation. Peptide was dissolved at 50 μM in 20 mM Tris-HCl pH 7.4 and incubated at room temperature without agitation for 168 h before TEM analysis. Scale bar: 100 nm.

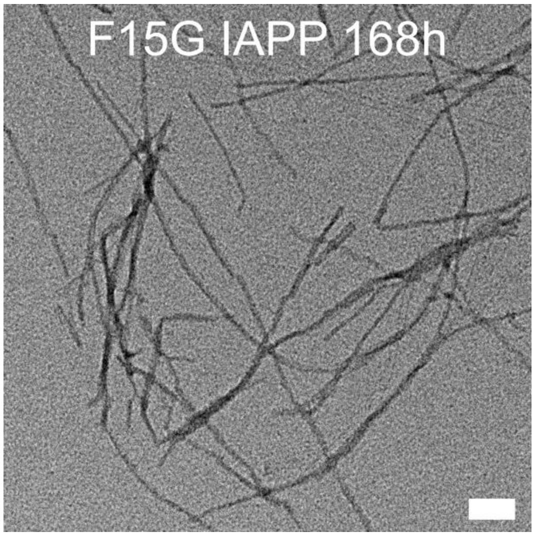

Figure S12. TEM image of F15G IAPP after 168h incubation. Peptide was dissolved at 50 μM in 20 mM Tris-HCl pH 7.4 and incubated at room temperature without agitation for 168 h before TEM analysis. Scale bar: 100 nm.

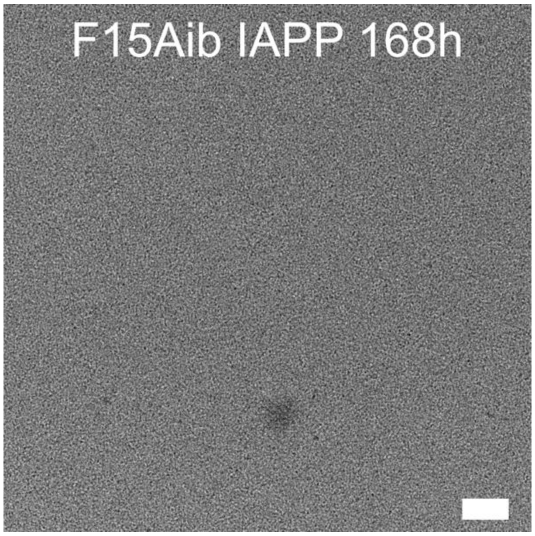

Figure S13. TEM image of F15Aib IAPP after 168h incubation. Peptide was dissolved at 50 μM in 20 mM Tris-HCl pH 7.4 and incubated at room temperature without agitation for 168 h before TEM analysis. Scale bar: 100 nm.
